# Galectin-9 Alleviates LPS-Induced Preeclampsia-Like Impairment in Rats via Switching Decidual Macrophage Polarization to M2 Subtype

**DOI:** 10.3389/fimmu.2018.03142

**Published:** 2019-01-10

**Authors:** Zhi-Hui Li, Li-Ling Wang, Hong Liu, Kahinho P. Muyayalo, Xiao-Bo Huang, Gil Mor, Ai-Hua Liao

**Affiliations:** ^1^Family Planning Research Institute, Center for Reproductive Medicine, Tongji Medical College, Huazhong University of Science and Technology, Wuhan, China; ^2^Reproductive Immunology Unit, Department of Obstetrics Gynecology and Reproductive Science, Yale University School of Medicine, New Haven, CT, United States

**Keywords:** Galectin-9, Tim-3, pre-eclampsia, decidual macrophage, polarization

## Abstract

Dysfunction of decidual macrophages (DMs) is considered a critical event in the pathogenesis of pre-eclampsia (PE). T cell immunoglobulin mucin 3 (Tim-3) is an important negative regulatory molecule that induces immune tolerance by interacting with its ligand Galectin-9 (Gal-9) and thus modulating function of various immune cells, including macrophages. However, the regulatory effects of Tim-3/Gal-9 signaling on DMs polarization and its role in PE remain unclear. In this study, we established a PE-like rat model by administering 1.0 μg/kg lipopolysaccharide (LPS) to normal pregnant Sprague-Dawley rats via the tail vein at embryonic day 5 (E5). Apart from the pre-eclamptic manifestations, increased M1 subtype and decreased M2 subtype were observed at the maternal-fetal interface, as well as increased pro-inflammatory cytokines (TNF-α and IL-1β) and reduced anti-inflammatory cytokines (TGF-β and IL-10). Moreover, the expression of Tim-3 in DMs and that of Gal-9 at the maternal-fetal interface were reduced. After administration of recombinant Galectin-9 (rGal-9) protein, we found that liver and renal injuries and maternofetal placental functional deficiency, including inadequate trophoblast cells invasion, impaired spiral artery remodeling and fetal capillary development, were reversed. In addition, the polarization of DMs was inclined to M2 subtype, which was similar to the polarization of DMs in the control rats but contrary to the PE-like rats. Interestingly, at E9, the expression of Tim-3 in DMs and that of Gal-9 at the maternal-fetal interface were significantly increased in the rGal-9 protein intervention group. Taken together, our findings show that administration of rGal-9 protein can alleviate the PE-like rat manifestations induced by LPS. This finding may be related to the activation of the Tim-3/Gal-9 signaling pathway, which promotes DMs polarization dominantly shifting to M2 subtype. Moreover, upregulation of Tim-3 in DMs and Gal-9 at the maternal-fetal interface at E9 suggests that Tim-3/Gal-9 pathway may play some important roles in early pregnancy and even embryo development.

## Introduction

Pre-eclampsia (PE) is a pregnancy-specific, immune-mediated syndrome characterized by the onset of high blood pressure and often accompanied by increased urine protein after 20 weeks of pregnancy (1), although now proteinuria is no longer required for diagnosis according to the guidelines of the *American College of Obstetricians and Gynecologists* (2). Prevalence of PE is estimated to be approximately 5~8% of all pregnancies globally and may be higher in some developing countries. PE is a primary cause of maternal and prenatal mortality and morbidity (3). The exact pathogenesis is still not well-addressed, although considerable research has been conducted to explain its etiology. As PE is a life-threatening disease and lacks effective treatment, there is a pressing need to understand its pathogenesis and provide effective therapy for protecting both mothers and babies.

During normal mammalian pregnancy, maternal immunomodulation allows the homograft-fetus and mother to peacefully coexist without rejection by the immune system, which may be the consequence of the interaction of various types of immune cells (4). The immune cells in the decidua mainly consist of decidual natural killer (dNK) cells (approximately 70%), decidual macrophages (DMs, approximately 20%), and T cells (approximately 10%) (5). Various immune cells and cytokines secreted by these cells are involved in regulating trophoblast invasion, angiogenesis, and spiral artery (SA) remodeling and inducing immune tolerance (5). Therefore, precise immunomodulation is critical for a healthy pregnancy. Considerable evidences indicate that locally aberrant immune responses at the maternal-fetal interface are related to the pathogenesis of some adverse pregnancy outcomes, such as recurrent pregnancy loss (RPL), intrauterine growth restriction, and PE (6, 7). Abnormal macrophage polarization (8, 9), dysfunction of NK cells (10), and imbalance between T helper (Th)1/Th2/Th17/Treg (11) in complicating pregnancy have been well-described by our group and others. However, most studies previously focused on the characteristics of NK and T cells at the maternal-fetal interface, and the potential roles and regulation of macrophage polarization in PE have not been well-explored.

Macrophages are the “bridge” between adaptive immunity and innate immunity, and they have critical roles in placentation and immunoregulation (12). Elaborated balance between M1 and M2 is essential for establishment and maintenance of healthy pregnancy (13). Imbalance of M1/M2 macrophages has been considered one of the causes of pregnancy-related diseases, such as PE, fetal growth restriction (FGR) and premature birth (14, 15). Studies have shown that M1-like subtype macrophages are increased in PE (16, 17). However, most studies simply found this imbalance of M1/M2 macrophages or addressed its involvement in the pathogenesis of pregnancy-related diseases, whereas the regulation of macrophage polarization in decidua is still unclear. Investigations with DMs could help to clarify their roles in healthy pregnancy and even further the development of new therapeutic strategies for pathologies of pregnancy.

T cell immunoglobulin mucin-domain 3 (Tim-3) is a negative regulator of costimulatory signaling molecules mainly expressed on terminally differentiated Th1 cells but not on Th2 cells, and it involved in autoimmune responses, immune tolerance, anticancer and antiviral immune evasion (18, 19). Tim-3 expression was identified on a variety of immune cells at the maternal-fetal interface, including CD8^+^ T cells, NK cells, Th17 cells, Treg cells, dendritic cells, monocytes, and macrophages (20, 21). Emerging studies have suggested that Tim-3 can regulate macrophage polarization. In sepsis, blocking Tim-3 promoted M1 macrophage polarization bias (22). When the expression of Tim-3 was upregulated, macrophage polarization deviated to the M2 subtype and then promoted the growth of tumors (21). In another study, Tim-3 overexpression attenuated inflammatory bowel disease by inhibiting the polarization of pathogenic, pro-inflammatory M1 macrophages (20). At the maternal-fetal interface, blockade of Tim-3 resulted in an accumulation of inflammatory granulocytes and macrophages and upregulation of pro-inflammatory cytokines (23). However, whether aberrant macrophage polarization was modulated by Tim-3 in PE is not yet clear.

Galectin-9 (Gal-9), as a ligand of Tim-3, is an important regulator of Th1 immunity and tolerance induction when interacting with Tim-3 (18, 24). Gal-9 is broadly expressed or secreted by various cells, including trophoblast cells (25). The Tim-3/Gal-9 pathway can induce cell death, especially exhaustion or apoptosis of effector T cells, and subsequently establish immune tolerance (19). An altered Tim-3/Gal-9 pathway, which has been identified in PE, leads to an enhanced systemic inflammatory response, including the activation of Th1 lymphocytes that participate at the onset of PE (26). Recent data showed that the Tim-3/Gal-9 pathway was crucial in regulating the function of dNK, which was critical for maintaining a normal pregnancy (27). However, whether DMs polarization can be regulated by the Tim-3/Gal-9 pathway is largely unknown. Here, we hypothesized that the aberrant maternal immune response may be triggered by adverse macrophage polarization in PE, which may be mediated by the altered Tim-3/Gal-9 pathway.

In our study, using a PE-like rat model induced by lipopolysaccharide (LPS), we demonstrate that abnormal macrophage polarization was involved in the pathogenesis of PE. Decreased Tim-3 expression in DMs may contribute to macrophage polarization to M1 subsets accompanied by insufficient trophoblast cell invasion and impaired SA remodeling in PE-like model rats. Interestingly, administration of recombinant Gal-9 protein can reverse the damage induced by LPS, possibly by upregulating Tim-3 expression in DMs, and the reversal effect can be observed at embryonic day 9 (E9), suggesting that Tim-3 may play important roles in early pregnancy or embryo development. However, the exact mechanism underlying reversal effects needs further investigation.

## Materials and Methods

### Animals and Experimental Design

All procedures and protocols were reviewed and approved by the Institutional Animal Care and Use Committee of Tongji Medical College, Huazhong University of Science and Technology, Wuhan, China. All animal care and use was performed in accordance with the approved guidelines of the Institutional Animal Care and Use Committee of Tongji Medical College.

**Figure 1:**
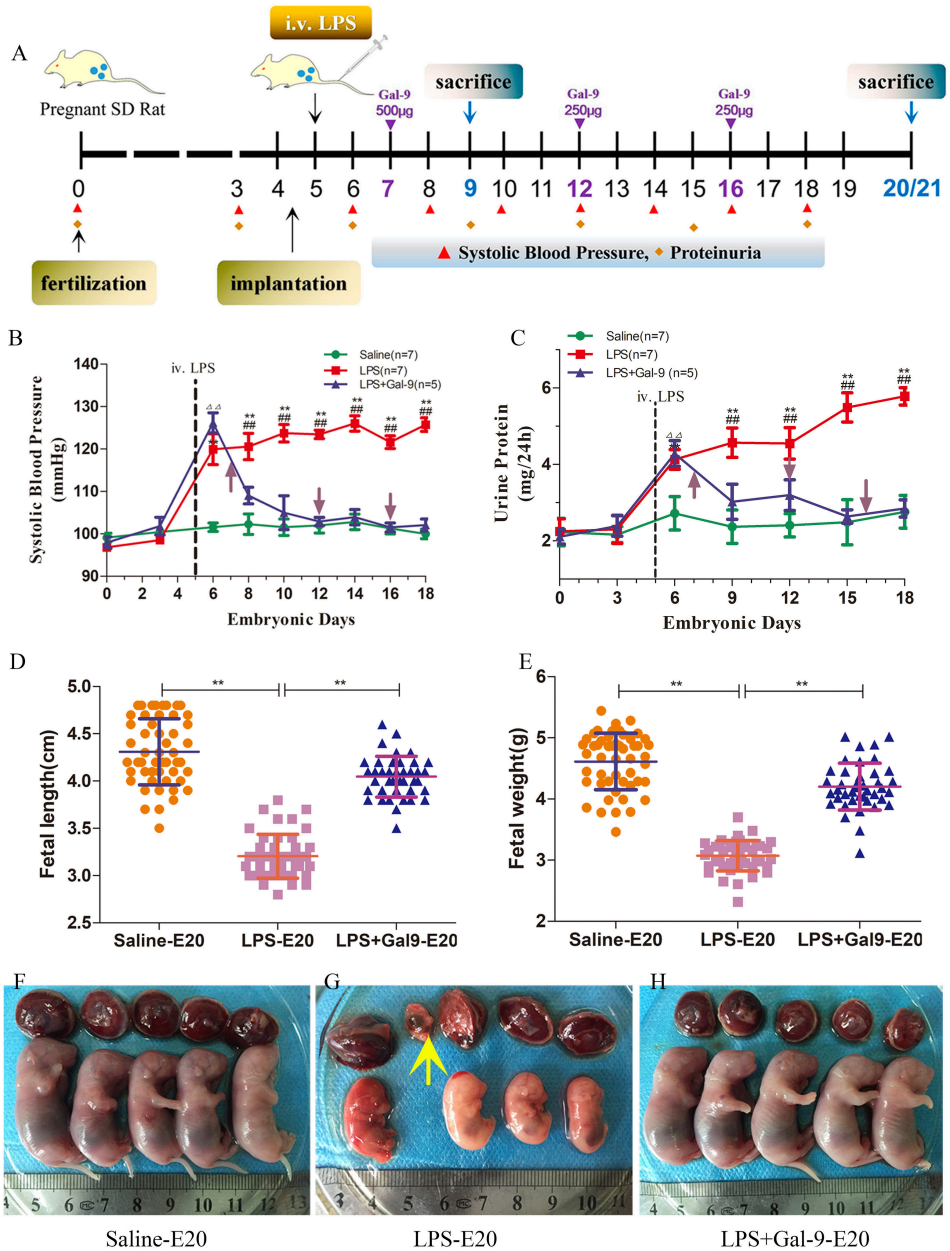
Schematic diagram of the animal experimental design and the mean SBP, 24 h urinary protein, and fetal development in each group. **(A)** Presence of vaginal spermatozoa confirmed successful pregnancy and was designated E0. Normal pregnant rats were injected with LPS via the tail vein at E5 to establish a PE-like rat model. The control group was injected with saline at the same time. Gal-9 recombination protein was administered to the PE-like rats by intraperitoneal injection at E7, E12, and E16. **(B)** SBP was monitored at E0, 3, 6, 8, 10, 12, 14, 16, and 18 and **(C)** 24 h urinary protein was measured at E0, 3, 6, 9, 12, 15, and 18. Saline, *n* = 7; LPS, *n* = 7; LPS+Gal-9, *n* = 5. Data are expressed as the mean ± SEM. ^ΔΔ^*p* < 0.01 LPS+Gal-9 vs. saline group at E6; **p < 0.01 LPS vs. saline group on the corresponding E; ^##^*p* < 0.01 LPS vs. LPS+Gal-9 group on the corresponding E. **(D–H)** Fetal and placental development in each group. **(D)** Fetal length and **(E)** fetal wet weight in different group, **p < 0.01. **(G)** Embryo absorption (yellow arrow).

Sprague-Dawley (SD) rats were purchased from the Animal Center of Tongji Medical College. Female rats weighing approximately 200 ~ 250 g were raised in a light- and humidity-controlled room (12: 12 h) with free access to food and water. After being allowed to acclimatize for 1 week, they were mated overnight with healthy male SD rats at a 2:1 ratio. As shown in Figure 1A, the presence of vaginal spermatozoa was used to confirm successful pregnancy and was designated as E0. Normal pregnant SD rats were randomly divided into three groups: (1) seven rats were injected with 1.0 μg/kg LPS/body weight (LPS, Sigma-2880, St. Louis, MO, USA) via the tail vein at E5 to create the PE-like model according to the methods and dosages from literatures (28–31), and the dams were sacrificed at E20; (2) ten rats were injected with the same dosages of LPS at E5 and further treated with recombinant Gal-9 protein (Chimerigen, San Diego, CA, USA) at indicated times, 500 μg at E7, 250 μg at E12 and 250 μg at E16, respectively; and the dams were sacrificed at E9 (five rats) and E20 (five rats); and 3) seven rats were given saline as controls.

### Detection of Blood Pressure and Urinary Protein and Evaluation of Offspring Development

As shown in Figure 1A, the systolic blood pressure (SBP) (08:00 a.m.~12:00 a.m.) of each rat was monitored prior to pregnancy and every 2 or 3 days after pregnancy with the ZH-HX-Z animal non-invasive blood pressure measuring system (Zhenghua Biological Instrument and Equipment Limited Corporation, Anhui Province, China). SBP was assessed continuously 3 times, and 5 continuous values with variation < 6 mmHg were averaged to define maternal SBP.

The urine of each rat was collected prior to pregnancy and every 2 or 3 days from E0 ~ E20 (09:00 am ~ 09:00 am next day) individually in metabolic cages, without any food but with free access to water (Figure 1A). Urinary protein was measured with a BCA protein assay kit (Beyotime Biotechnology, P0010S, China).

After the rats were sacrificed, the numbers of visible and absorbed pups were recorded. The placental and fetal developments were compared.

### Tissue Collection

At E20, the rats were sacrificed using pentobarbital sodium anesthesia. The livers and kidneys were collected rapidly and fixed in 4% paraformaldehyde. Whole implantation sites, the placenta with its associated mesometrial triangle (MT) and decidual tissues were carefully separated. Some were fixed in 4% paraformaldehyde, and the others were stored in −86°C refrigerators prepared for mRNA and protein detection. Section tissues were fixed at least 24 h at 4°C, dehydrated through increasing alcohol concentration and then embedded with paraffin.

### Histology of Liver, Kidney and Implantation Site

For histological analysis, 3 μm thick paraffin sections of rat liver tissues and transverse sections of kidney were cut. Liver slices were stained with H&E, and kidney slices were stained with H&E and PAS using standard protocols.

The implantation sites were cut into 3 μm thick paraffin sections vertical to the mesometrial-fetal axis. Selected sections containing the central maternal arterial channel were stained with PAS, which is a fibrinoid tissue marker.

Histological evaluation of H&E and PAS staining were performed on 4 ~ 6 random fields photographed at different magnifications to acquire images using an Olympus BX51 microscope and Olympus DP70 manager (Japan).

### Immunohistochemistry (IHC)

The 3 μm thick paraffin sections of placenta vertical to the uterine-fetal axis were dewaxed in xylene, rehydrated with decreasing concentrations of ethanol, and then washed in PBS. Antigens were unmasked by microwaving in 10 mmol/L citrate buffer at a pH of 6.0 (15 min). Endoperoxidase activity was blocked by 3% H_2_O_2_ in methanol, and non-specific sites were blocked with 5% normal serum. The primary antibodies of laminin and Gal-9 were incubated at 4°C overnight. After incubation with secondary antibody, color development was measured using 3, 3'-Diaminobenzidine tetrahydrochloride, followed by staining the nucleus with hematoxylin. Then, the sections were dehydrated through increasing concentrations of ethanol and xylene. Slides were created on 4 ~ 6 random fields photographed at different magnifications to acquire images using an Olympus BX51 microscope and Olympus DP70 manager (Japan). The primary and secondary antibodies used in the experiments were shown in Supplementary Table S1.

### Immunofluorescence (IF) Staining of the Implantation Site and Image Analysis

Whole implantation sites fixed in 4% paraformaldehyde at 4°C (4 ~ 6 h) were dehydrated through increasing sucrose and repaired in 30% sucrose overnight at 4°C and subsequently embedded in optimal cutting temperature compound (OCT, Sakura-4583, USA) and finally fast-frozen. Then, 4 μm-thick serial sections were cut.

After antigen retrieval, the sections were incubated in blocking buffer (5% bovine serum albumin and 0.3% Triton-X 100 in PBS) for 30 ~ 45 min at room temperature. Primary antibodies were added, and slides were incubated at 4°C overnight. Secondary antibodies were added and incubated at room temperature (approximately 25°C, 2 h), followed by staining the nucleus with 4′, 6-diamidino-2-phenylindole. All IF staining was performed in the dark. Slides were created with 4 ~ 6 random fields photographed at × 400 magnification to acquire images using an Olympus BX51 microscope and Olympus DP70 manager (Japan). The fluorescence intensity was quantified, and colocalization was analyzed by ImageJ software (Java 1.8.0_112, NIH, USA). The primary and secondary antibodies used in the experiments were shown in Supplementary Table S1.

### Quantitative Real-Time PCR (qRT-PCR)

Total RNA was extracted with TRIzol reagent (Life technologies, CA, USA) following the manufacturer's instructions. An equal amount of total RNA (1 μg) was treated with gDNA Eraser reagent to eliminate potential genomic DNA and then used for cDNA synthesis (Takara Bio, Shiga, Japan). qRT-PCR amplification analysis was performed with 2 μl of cDNA using a SYBR® Premix Ex TaqTM II kit (Takara Bio, Shiga, Japan) on a LightCycler®96 (Roche, Basel, Switzerland). Relative mRNA levels to calibrator were computed using the 2^−ΔΔCT^ method and β-actin was used for normalization. Primers used in this study were shown in Supplementary Table S2.

### Preparation of Single Cell Suspensions and Flow Cytometry (FCM) Analysis

Whole implantation sites were removed from the rat uteri at E9 and E20, respectively, and washed with pre-cold PBS. Then the deciduas were separated, sliced with scissors and finally digested in DMEM containing 1.0 mg/ml collagenase Type IV (Gibco, Grand Island, NY, USA) and 100 U/ml DNase I (Sigma-Aldrich, Saint Louis, MO, USA) for 1 h in a constant temperature oscillator at 37°C. The mixture substance was filtered via 53 μm nylon cell strainer. Then the single cells were collected, centrifuged and resuspended in erythrocyte lysate (Saiweier Biotechnology, Wuhan, China) at 4°C for 15 min. After centrifugation, the cells were collected for FCM analysis.

The following antibodies were used: FITC-conjugated mouse anti-rat CD45 (clone: OX1, 11-0461, eBioscience, San Diego, CA, USA), RPE-conjugated mouse anti-rat CD68 (MCA341PE, AbD Serotec) and Alexa Fluor® 647- conjugated mouse anti-rat CD163 (MCA342A647, AbD Serotec). The experiments were performed according to the manufacturer's instructions. The labeled cells were analyzed with LSR II Flow Cytometer (BD Biosciences, San Jose, CA, USA) and Flow Jo software (Tree Star, Ashland, OR, USA) was used for data analysis.

### Western Blot Analysis

In preparation for Western blot analysis, the whole implantation site was dissected, and only its associated MT and decidual tissues were maintained as far as possible. The tissues were homogenized with RIPA buffer (50 mM Tris pH 7.4, 150 mM NaCl, 1 mM EDTA, 1% Triton X-100, 1% sodium deoxycholate, 0.1% SDS) containing protease inhibitors. Protein concentrations were measured with a BCA protein assay kit (Beyotime Biotechnology, P0010S, China) and boiled in 5 × SDS-PAGE loading buffer. After separation by SDS-PAGE and transfer to a polyvinylidene difluoride membrane, immunoblotting was blocked with 5% skim milk (BD, USA) and primary antibodies against CD68, CCR7, Arg1, Tim-3, Gal-9 and β-actin were added. The membranes were washed with TBS/T and incubated with secondary antibodies (Supplementary Table S1), followed by an enhanced chemiluminescence detection kit (Beyotime Biotechnology, P0018, China). Relative protein levels were acquired by a Universal Hood 2 Electrophoresis Imaging Cabinet, Chemi DOC (Biorad, USA) and quantified by Image J software. Densitometry values were normalized to totalβ-actin.

### Statistical Analysis

Data are presented as mean ± SEM. Differences between means were analyzed using Student's *t*-test or the Mann-Whitney *U*-test as applicable. One-way ANOVA was used for multiple comparisons. Significance was defined as *P* < 0.05. Statistical analyses were performed using Statistical Package for Social Science for Windows (Version 22.0 software, SPSS Inc., Chicago, IL, USA) the GraphPad Prism software, version 7.

## Results

### Gal-9 Ameliorated Hypertension and Proteinuria in LPS-Induced PE-Like Rats

To investigate the potential roles of Gal-9 in PE, we established a PE-like rat model that mimics some of the symptoms observed in patients with PE. According to previously described methods and dosages (28–31), tail vein injection of LPS (1.0 μg/kg at E5) induces a significant increase in SBP, higher levels of proteinuria, and adverse pregnancy outcomes compared to control pregnant rats injected with saline.

As shown in Figure 1 and Supplementary Table S3, the control group (saline) exhibited stable SBP throughout the entire gestational period. However, in the rats treated with LPS, we observed significantly increased SBP post injection (at E6), which remained high throughout the pregnancy (Figure 1B). However, the SBP wasn't remarkably increased in non-pregnancy rats injected with LPS (Supplementary Figure S1). Interestingly, a single administration of Gal-9 (500 μg) at E7 to the rats treated with LPS (at E5) was able to reverse the effect of LPS and decrease SBP (Figure 1B). Additional administrations of Gal-9 (250 μg) at E12 and E16, respectively, maintained the SBP at the same levels as those observed in the control (saline) group.

Next, we evaluated the levels of proteinuria, a clinical marker of renal malfunction associated with PE (3). As shown in Figure 1C and Supplementary Table S4, administration of LPS is associated with a significant increase in proteinuria 24 h post treatment compared to the control (saline), indicative of the presence of renal dysfunction. However, this change could not be observed in non-pregnancy rats injected with LPS (Supplementary Figure S1). Similar to the effect described for SBP, administration of Gal-9 at E7, E12 and E16, respectively, restored proteinuria to the normal levels as observed in the control (saline) group.

In addition to evaluating changes in SBP and proteinuria, we also determined whether the treatment with LPS could affect placental and fetal development. As shown in Figures 1D–H and Supplementary Table S5, fetal length, fetal wet weights, and placental shape and color determined for all the three groups were recorded when the rats were sacrificed at E20. The fetuses from the rats treated with LPS alone showed restricted development, characterized by small size (Figure 1D), low weight (Figure 1E) and presence of limb deformation (Figure 1G). However, that was not the case in the group of rats treated with Gal-9, which was able to protect the fetus from the detrimental effects of LPS (Figure 1H). These findings suggested that in rat model, Gal-9 administration could provide a protective effect for the fetus and the mothers.

The placentas from the rats treated with LPS alone were characterized by irregular shape and bleak color suggestive of placental ischemia (Figure 1G). However, these changes were not found in the placentas obtained from the rats treated with Gal-9 throughout the pregnancy, which looked similar to those of the control (saline) group (Figures 1F,H).

### Gal-9 Prevented Liver and Renal Injury in LPS-Induced PE-Like Rats

**Figure 2:**
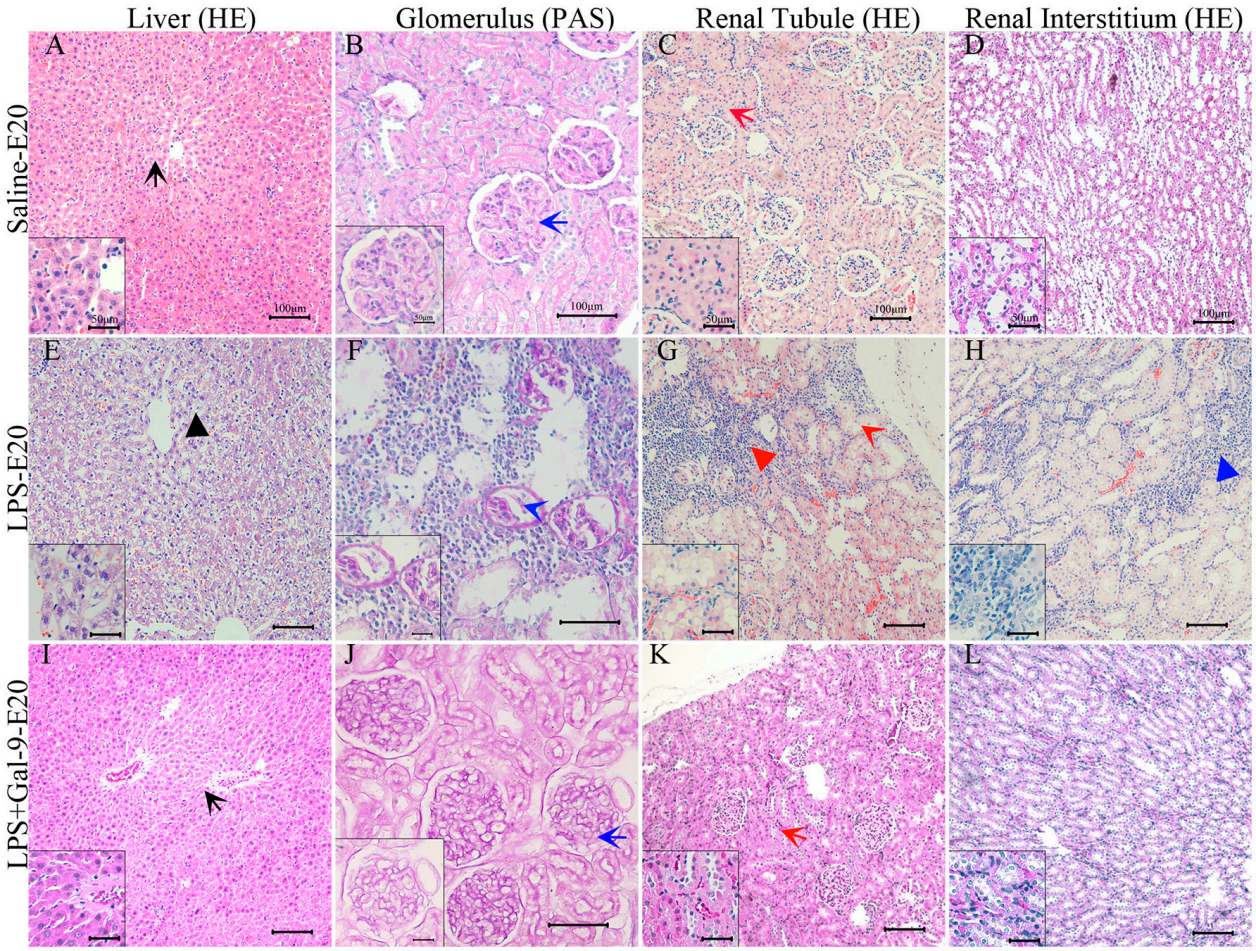
Morphological features of the liver and kidney in the different groups. H&E staining of liver **(E)** and kidney tissue **(F–H)** from a PE-like rat showed hepatocyte balloon degeneration and spotty necrosis (black short arrow, **E**), renal tubular epithelial cell collapsed and swollen (red long arrow, **G**), inflammatory cell infiltrates in the cortex (red short arrow, **G**) and interstitial tissue (blue short arrow, H). PAS staining of kidney tissue from the PE-like rats showed glomerular swelling and even atrophy and mesangial extracellular matrix expansion (blue long arrow, **F**). After Gal-9 recombination protein intervention, H&E and PAS staining of liver **(I)** and kidney tissues **(J–L)** were basically normal without obvious pathological changes, which was similar to normal pregnancy **(A–D)**. Scale bars: 100 μm, 50 μm.

Our next objective was to characterize the mechanism by which LPS injection during pregnancy promotes hypertension and proteinuria. Thus, we collected liver and kidney samples from the mothers receiving saline, LPS, and LPS+Gal-9 and examined histological changes by H&E or PAS staining. H&E staining of liver tissues showed that administration of LPS induced hepatocyte steatosis, inflammation (leukocyte infiltration), ballooning degeneration of the hepatocytes and spotty necrosis (Figure 2E). None of these changes were observed in the control group (Figure 2A). Evaluation of the renal tissues revealed significant alterations in glomerular morphology characterized by mesangial extracellular matrix expansion (percentage of PAS^+^ area increased, Figure 2F) along with a significant glomerular swelling and atrophy (Figures 2F–H). In addition, the proportion of renal tubular swelling accompanied by collapsing tubular epithelial cells was greater in the LPS-treated group (Figure 2G). We also observed local inflammatory infiltrates in the renal interstitial tissue (Figure 2H). In contrast, the rats treated with Gal-9 were protected from the deleterious effect by LPS. We observed few liver and renal injuries (Figures 2I–L), and their structural characteristics were similar to the samples obtained from the control (saline) group (Figures 2A–D).

### Gal-9 Reversed LPS Induced Maternofetal Placental Function Deficiency

**Figure 3:**
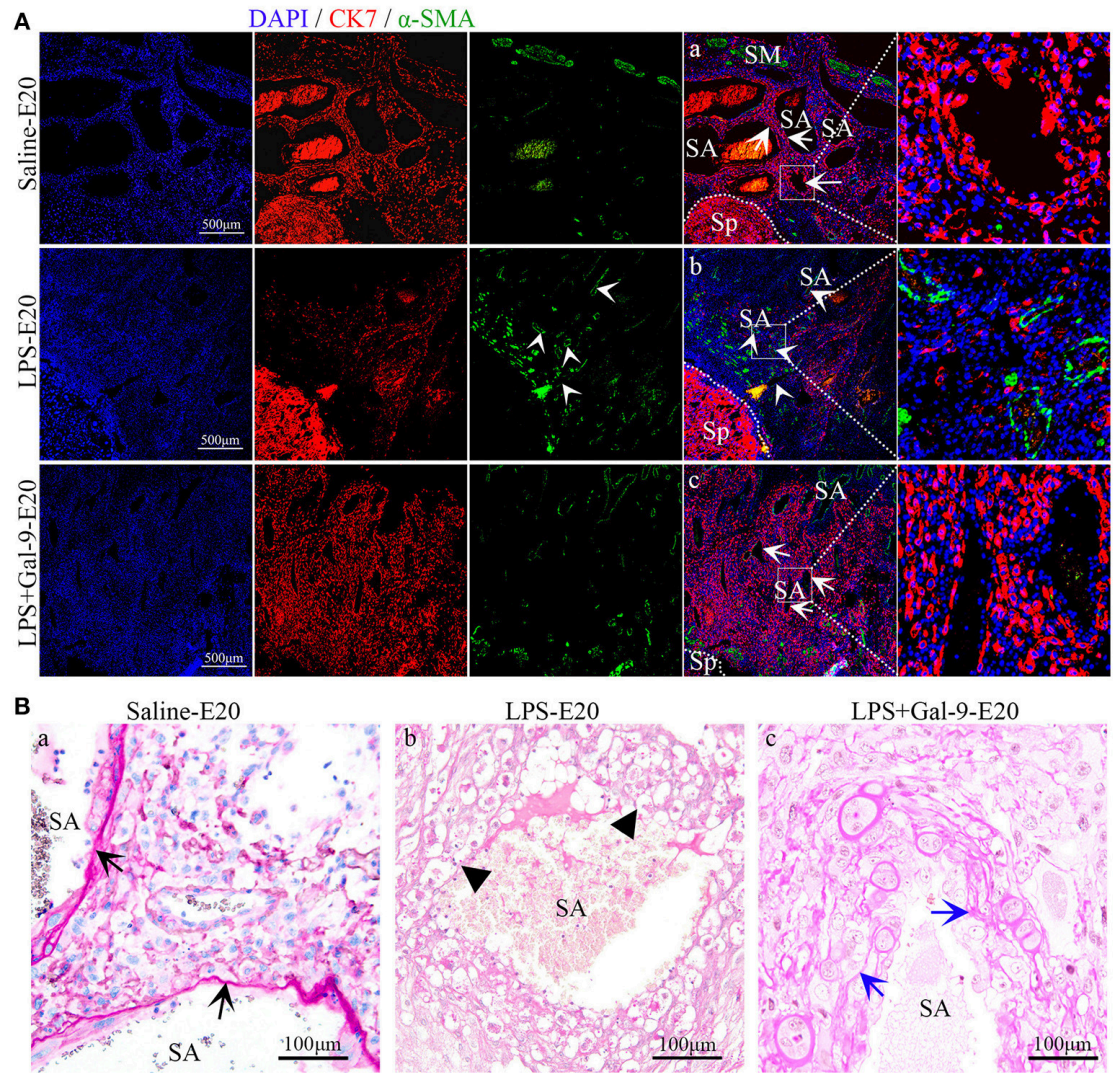
Trophoblast invasion and SA remodeling in different groups. **(A)** There were fewer trophoblasts (CK7 staining, red) and more vascular smooth muscle (α-SMA staining, green) in the LPS-treated group (white short arrow, b). In the Gal-9 intervention group, trophoblast invasion, and SA remodeling were reversed (white arrow, c), similar to the control pregnancy group (white arrow, a). Scale bars: 500 μm. **(B)** PAS staining of fibrinoids in the SA of the mesometrial triangle in different groups. In the control pregnancy group, the fibrinoid wall was obviously integrated (black arrow, a). In comparison, the fibrinoid wall almost disappeared in the LPS-treated group (black short arrow, b). In the Gal-9 intervention group, the fibrinoid wall was formed (blue arrow, c). SA, spiral artery. Scale bars: 100 μm.

To elucidate how LPS affected the development of the placenta and fetus, we evaluated the histologic characteristics of the placenta samples by morphometry and immunofluorescence (IF) for markers of placenta function. IF of pan-cytokeratin 7 (CK7 staining), alpha-smooth muscle actin (α-SMA staining) and PAS staining of the entire area of the MT showed marked structural alterations in the placenta from the LPS-treated rats, mainly characterized by fewer trophoblast cells, more vascular smooth muscle but less fibrinoid wall (PAS staining), suggestive of impaired SA remodeling. However, there was significantly greater trophoblast invasion and more fibrinoid wall, but obviously less vascular smooth muscle, in the Gal-9 group and the control group compared with the LPS treated group (Figure 3, Supplementary Figure S2A).

**Figure 4:**
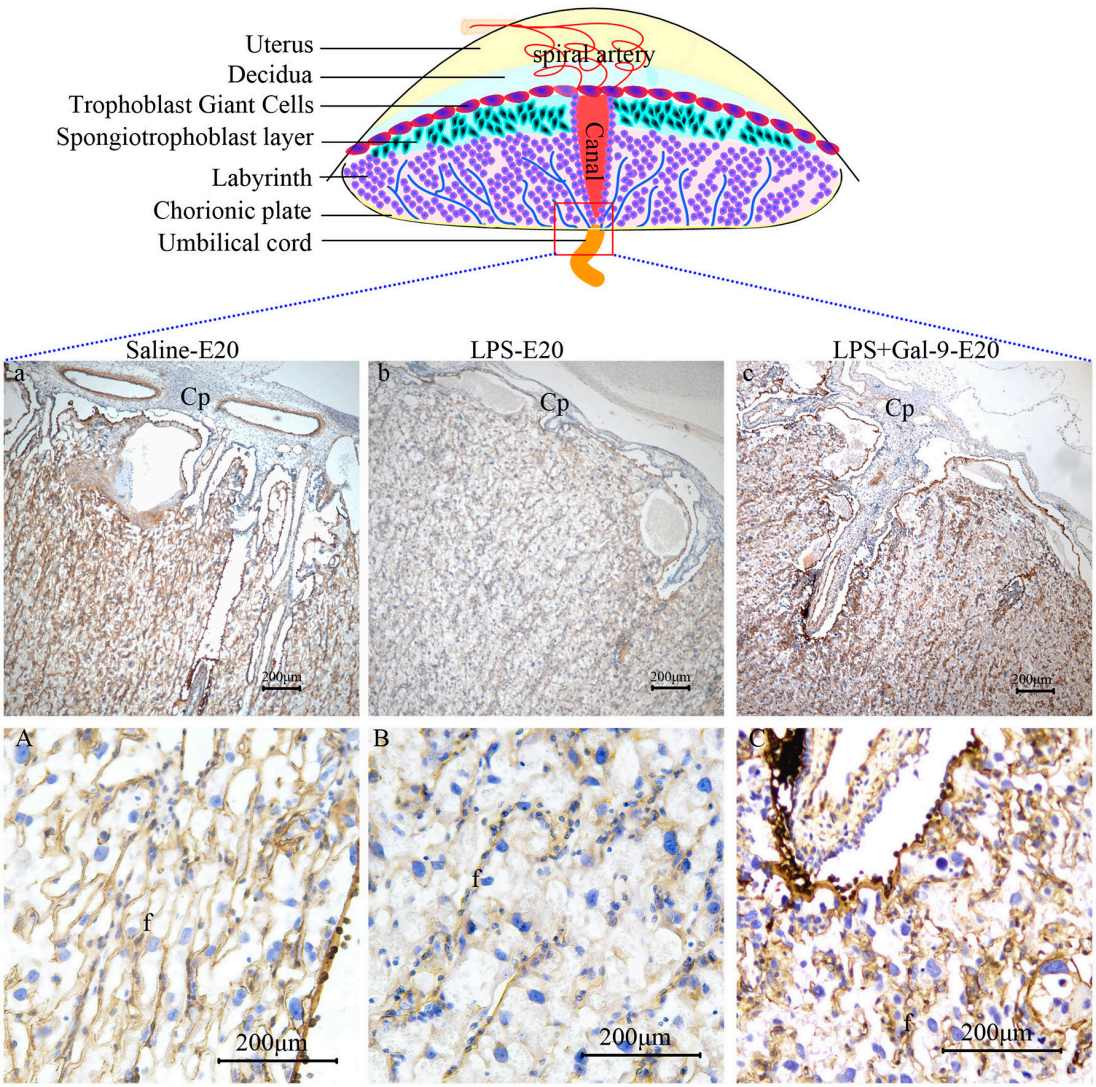
Immunohistochemistry for laminin in the labyrinth of different groups. There were fewer branches from Cp (b), fewer fetal capillaries and narrowed lumen **(B)** in the labyrinth of LPS-treated group. In the Gal-9 intervention group, the branches from Cp increased (c) and the maternal-fetal vascular network fully developed **(C)**, which was similar to the control pregnancy group (a and **A**). Cp, Chorionic plate; f, fetal vessel. Scale bars: 200 μm.

To assess placental perfusion, we evaluated the expression of laminin, a marker of fetal blood vessels, by IHC in the labyrinth of placentas obtained from the pregnant rats at E20. In the placentas from the LPS-treated rats, we observed decreased expression of laminin and fewer branches from the fetal capillaries, indicative of an impaired maternal-fetal vascular network in the labyrinth, suggesting that deficient capillary development might be the cause of poor placental development. None of these abnormal changes were observed in the samples of rats that received LPS followed by Gal-9 treatment. Similar to the control group, the placentas in the LPS+Gal-9 group were characterized by the presence of elongated and highly branched capillaries suggestive of fully developed maternal-fetal vascular networks (Figure 4). These findings further suggested that Gal-9 might reverse LPS-induced maternofetal placental function deficiency.

### Gal-9 Suppressed the Polarization of DMs Biased to M1 but Promoted a Shift to M2

Macrophages in the decidua are characterized by an anti-inflammatory (M2) phenotype and are responsible for SA remodeling, trophoblast invasion and removal of apoptotic cells (32). However, macrophages have a high degree of plasticity, and their phenotype can change in response to dangerous signals by modifying their phenotype to a pro-inflammatory (M1) type (12, 16). We hypothesized that LPS drives macrophages toward the M1 phenotype, and the inflammation driven by these M1 macrophages might be responsible for the abnormal development of the placenta and fetus observed in the rats receiving LPS administration. Since Gal-9 has been shown to promote an M2 phenotype in an inflammatory bowel disease model (20), we postulated that Gal-9 could enhance the differentiation of M2 subsets. Consequently, our next objective was to i) determine the characteristics of DMs following LPS treatment and ii) establish whether administration of Gal-9 could modulate LPS-induced macrophage differentiation. Thus, we further investigated DMs polarization and the reverse effects of Gal-9 on the polarization of DMs.

**Figure 5:**
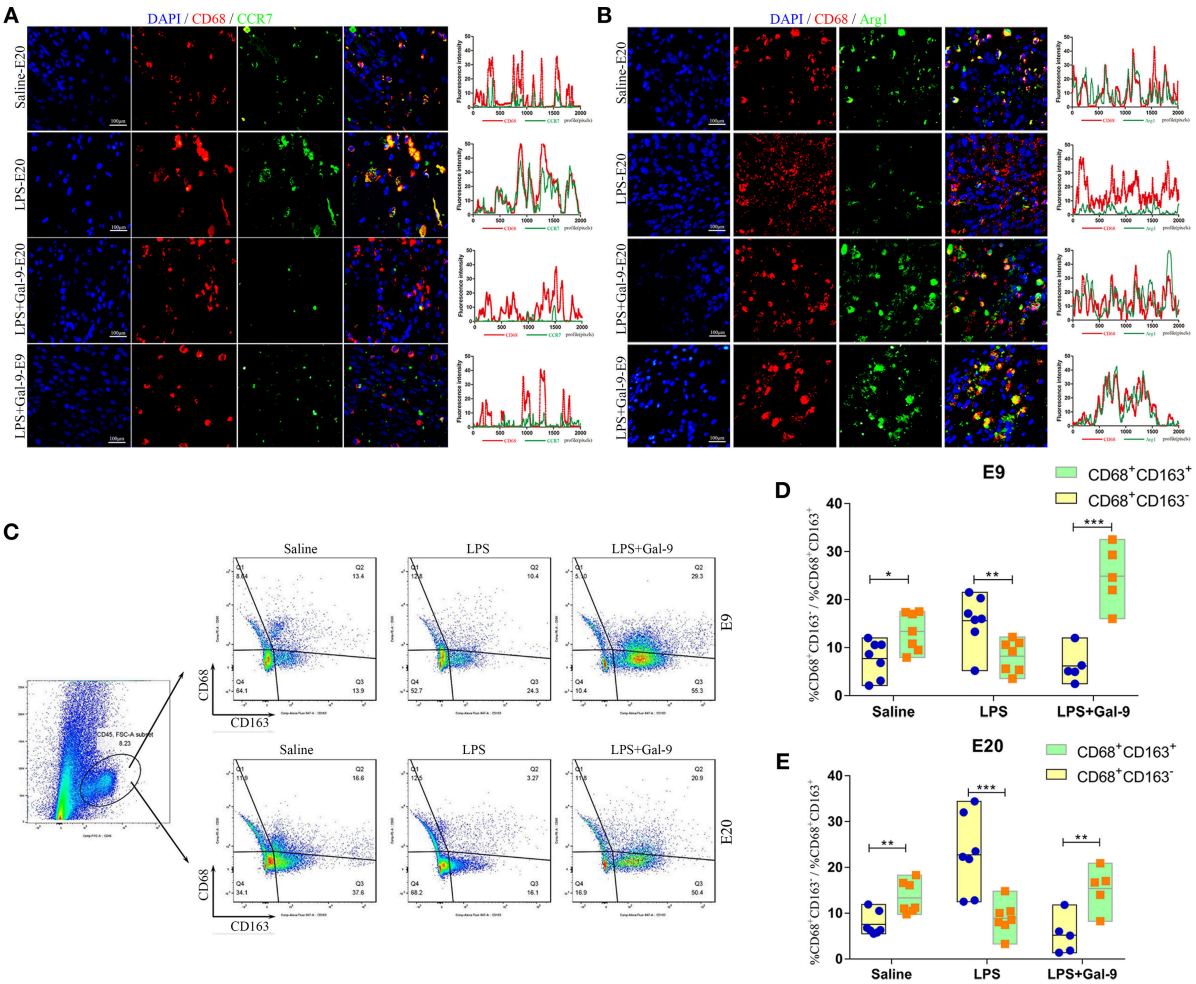
Gal-9 suppressed the polarization of DMs biased to M1 but promoted a shift to M2. **(A)** Immunofluorescence for CD68 and CCR7 in the decidua of different groups. The CCR7 expression on macrophages (CD68^+^CCR7^+^) in the decidua's of LPS-treated group was significantly increased. In the Gal-9 intervention group, M1 macrophages decreased, which was similar to the control pregnancy group. Scale bars: 100 μm. **(B)** Immunofluorescence for CD68 and Arg1 in the decidua of different groups. The Arg1 expression on macrophages (CD68^+^Arg1^+^) in the decidua's of LPS-treated group was significantly decreased. In the Gal-9 intervention group, M2 macrophages increased, especially at E9, which was similar to the control pregnancy group. Scale bars: 100 μm. **(C)** The percentages of M1 and M2 subsets in decidua were detected by FCM. A single-cell decidual suspension was obtained by enzymatic digestion. The total leukocyte population was identified in live cells by the expression of CD45, and then gated for identification of macrophage subsets by the expression of CD68 and CD163 (CD45^+^CD68^+^CD163^−^ and CD45^+^CD68^+^CD163^+^ represent M1 and M2 subsets, respectively). Contour plots are representative of three independent experiments. **(D,E)** Comparison of the percentages of M1 and M2 subsets at E9 and E20 from different groups (data are presented as mean ± SEM). *p < 0.05, **p < 0.01, ***p < 0.001.

First, we performed IF studies of CD68 (pan-macrophage marker), chemokine receptor 7 (CCR7, M1 marker) and Arginase 1 (Arg1, M2 marker) in the MT of the three groups at E9 and E20. As shown in Figure 5, we found CD68 expression in the MT of all three groups, but there were no significant differences among the three groups. In addition, CD68^+^CCR7^+^ (M1 subtype) in the decidua of the rats injected with LPS were significantly increased compared to that in the control group. However, in the group of rats treated with Gal-9, M1 subtype was decreased obviously and was similar to the control group (Figure 5A, Supplementary Figure S2B). To the contrary, CD68^+^Arg1^+^ (M2 subtype) was significantly decreased in the LPS-treated group and markedly increased after Gal-9 administration, nearly to the levels of control group (Figure 5B, Supplementary Figure S2B). It was worth noting that the increase in Arg1 expression started from E9 (early pregnancy in rats). This finding suggested that Gal-9 intervention may have a reverse effect in the early pregnancy of PE-like rat models.

In order to determine the percentage changes of M1 and M2 subsets in decidua, we performed FCM experiments. According to the previous literature (33), CD45^+^CD68^+^CD163^−^ and CD45^+^CD68^+^CD163^+^ were used to characterize M1 and M2 subsets in rats, respectively. As shown in Figures 5C–E, higher percentages of M1 subsets and lower percentages of M2 subsets were observed in the LPS-treated group when compared with the control group. However, after Gal-9 administration, M2 subsets were significantly increased while M1 subsets were overtly decreased.

**Figure 6:**
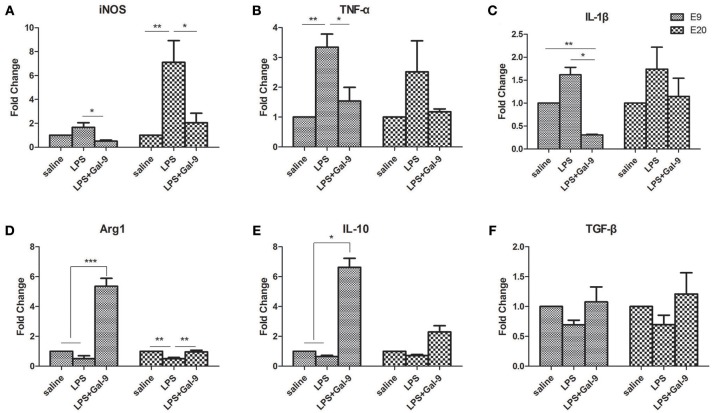
The mRNA expression levels of M1 and M2 markers, and pro-inflammatory and anti-inflammatory cytokines by qRT-PCR in different groups. **(A,D)** mRNA levels of iNOS (M1 subset marker) and Arg1 (M2 subset marker) in the placenta from the three groups. **(B,C)** mRNA levels of TNF-α and IL-1β (pro-inflammatory cytokines) and **(E,F)** mRNA levels of IL-10 and TGF-β (anti-inflammatory cytokines) in the placenta from the three groups. β-actin was used for normalization. Data are showed as the mean ± SEM, one placenta per rat, *p < 0.05, **p < 0.01, ***p < 0.001.

In addition, the mRNA levels of pro-inflammatory cytokines (TNF-α and IL-1β), anti-inflammatory cytokines (IL-10 and TGF-β), iNOS (a marker of M1 subset) and Arg1 (a marker of M2 subset) in MT of the different group were also assessed by qRT-PCR (Figure 6). In the LPS-treated group, the mRNA levels of iNOS, TNF-α and IL-1β were increased at E9, while decreased after the administration of Gal-9 (Figures 6A–C). The mRNA levels of Arg1 (*p* < 0.001) and IL-10 (*p* < 0.05) at E9 were significantly increased in the LPS+Gal-9 treated group as compared to the control (saline) and LPS-treated groups (Figures 6D,E). No significant difference was observed in the mRNA level of TGF-β among the three groups at both E9 and E20 (Figure 6F). At E20, the mRNA level of iNOS was increased in the LPS-treated group as compared with the control (*p* < 0.01) and LPS+Gal-9 (*p* < 0.05) groups. However, the mRNA level of Arg1 was obviously decreased in the LPS-treated group as compared with the control and LPS+Gal-9 groups (both *p* < 0.01). No significant difference was observed in the mRNA levels of TNF-α, IL-1β, and IL-10 among the three groups at E20.

Then, we further evaluated the CD68, CCR7, and Arg1 protein expressions in the MT at E9 and E20 by Western blot analysis. As shown in **Figure 8** and Supplementary Figure S3, we found that the results were consistent with the findings using IF. The protein expression of CD68 in the MT of all three groups was not significantly different (*p* > 0.05). In the PE model rats, LPS significantly increased CCR7 protein expression but decreased Arg1 protein expression compared to the control group (*p* < 0.05). Surprisingly, Gal-9 administration increased Arg1 protein expression, accompanied by decreased CCR7 protein expression in the MT at E20 and E9 (*p* < 0.05) compared to the LPS-treated group. However, there were no significant differences in the protein expressions of CCR7 and Arg1 between the LPS+Gal-9-treated and control groups (*p* > 0.05). Therefore, these results suggested that Gal-9 might suppress the polarization of DMs biased to M1 but promote a shift to M2.

### Gal-9 Inhibited the Down-Regulatory Effect of LPS on Tim-3 Expression in DMs

**Figure 7:**
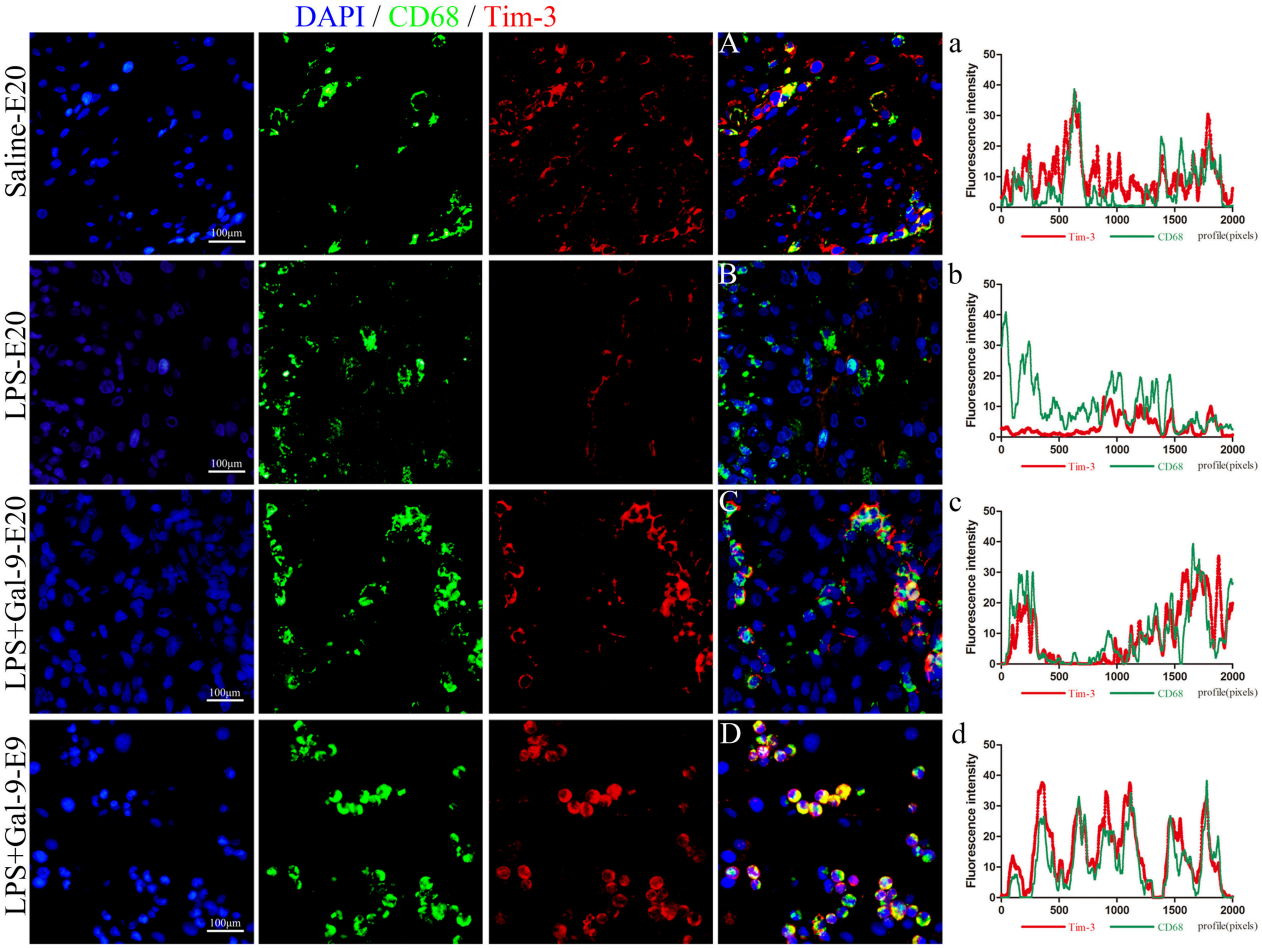
Immunofluorescence for CD68 (green) and Tim-3 (red) in the decidua of different groups. The expression of Tim-3 in DMs of the LPS-treated group at E20 was significantly decreased (**B** and b). In the LPS+Gal-9 treated group, the expression of Tim-3 was rescued (**C** and c, **D** and d), which was similar to the control (saline) group (**A** and a), especially at E9 (**D** and d). Pan macrophage marker CD68 is labeled in green, and Tim-3 labeled in red. DAPI labeled nuclei in blue. Scale bars: 100 μm.

**Figure 8:**
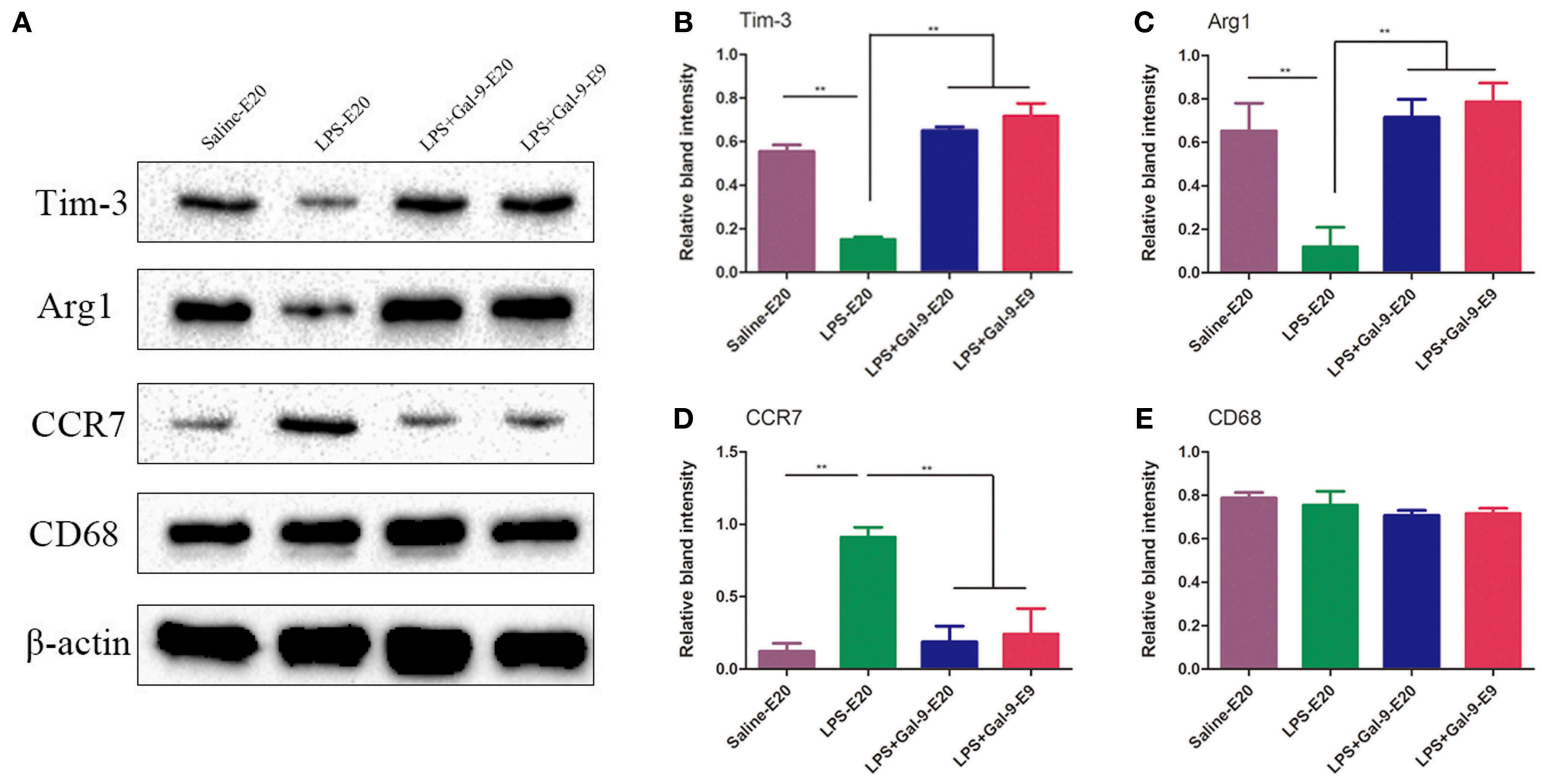
Western blotting for CD68, CCR7, Arg1 and Tim-3 in the decidua's in different groups. **(A)** The protein levels of Tim-3 (33kDa), Arg1 (35kDa), CCR7 (43kDa), and CD68 (37kDa) in the placenta were determined by western blotting and normalized to the level of β-actin (42kDa). **(B–E)** The quantified relative band intensity was determined by ImageJ. The results are shown as the mean ± SEM of three separate experiments, *p < 0.05, **p < 0.01.

To verify whether Gal-9 regulated the polarization of DMs by activating the Tim-3/Gal-9 pathway, the protein expression of Tim-3 in DMs was analyzed by IF and Western blot in the MT of the three groups. As shown in Figure 7, compared with those in the control group, CD68^+^Tim-3^+^DMs were significantly decreased in the MT of the LPS-treated group. After Gal-9 administration, Tim-3 expression in DMs obviously increased at both E9 and E20, similar to the control group. In addition, the expression of Tim-3 in DMs was mainly localized on the membranes of macrophages. Interestingly, similar to the expression of Arg1, we found that Tim-3 expression was upregulated at E9. In line with the findings from IF, Western blot results also showed that Tim-3 protein expression in the MT after Gal-9 treatment was highly increased compared to the LPS-treated group (*p* < 0.05), but there was no statistically significant difference between the control group and the Gal-9 treated group (*p* > 0.05). In addition, Tim-3 increased in the Gal-9-administered group at E9 and E20, but there were no significant differences between these two groups (*p* > 0.05) (Figure 8, Supplementary Figure S4).

### Exogenous Administration of Gal-9 Rescued the LPS-Induced Decrease of Gal-9 Expression at the Maternal-Fetal Interface

**Figure 9:**
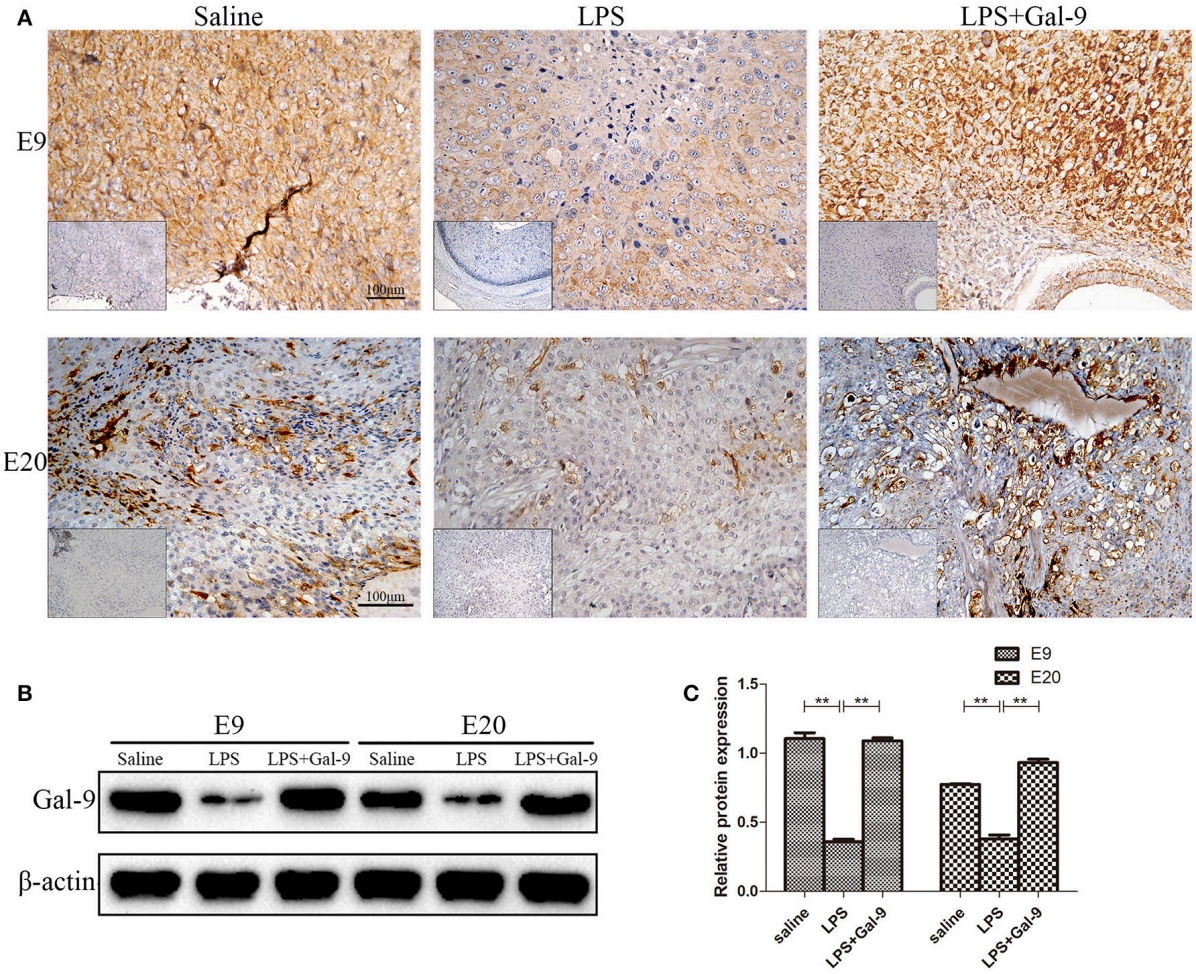
Immunohistochemistry and Western blotting for identification of Gal-9 at the implantation site of different groups. **(A)** Immunohistochemistry for Gal-9 at the implantation site of different groups. The pictures at the lower-left corner of each group are the negative controls. Scale bars: 100 μm. **(B)** The protein levels of Gal-9 (40 kDa) were determined by western blotting and normalized to the level of β-actin (42kDa). **(C)** The quantified relative band intensity was determined by ImageJ. The results are shown as the mean ± SEM of three separate experiments, *p < 0.05, **p < 0.01.

As a ligand of Tim-3, Gal-9 can be expressed or secreted by various cells, including trophoblast cells (25). By using IHC and Western blot, the expression and localization of Gal-9 protein were determined in MT of the three groups. As shown in Figure 9A, the Gal-9 expression was mainly localized at one pole of embryo implantation at E9, and in the spongiotrophoblast layer and decidua at E20. The Gal-9 protein levels were significantly decreased at E9 and E20 in the LPS-treated group compared to the control group (both *p* < 0.01), which could be rescued by exogenous administration of Gal-9 (*p* < 0.01) (Figures 9B-C, Supplementary Figure S5). However, Gal-9 expression in non-pregnant rat uteri was mainly localized on endometrial and glandular epithelium (Supplementary Figure S6). These results show that exogenous administration of Gal-9 could rescue the LPS-induced decrease of Gal-9 expression at the maternal-fetal interface.

## Discussion

The key findings in our study showed that an altered Tim-3/Gal-9 pathway may be involved in the pathogenesis of PE, and Gal-9 administration could alleviate the PE-like manifestations induced by LPS in rat models via regulating the polarization of DMs. Moreover, we improved the PE-like animal model based on methods previously described (28–31), by injecting 1.0 μg/kg LPS into normal pregnant SD rats at E5.

Interacting with toll-like receptor 4 (TLR4), LPS makes macrophages polarize to the M1 subtypes, which produce pro-inflammatory cytokines such as IL-1β, IL-6, IL-12, IL-23, and TNF-α (34). Previously published studies (28–31) have reported that LPS administration via the tail vein could establish a PE-like rat model, but the evaluation of these established models was far from perfect. Here, in our study, in addition to detecting the changes in blood pressure, urine protein and histopathology of the liver and kidney, we also evaluated trophoblast cell invasion, SA remodeling, and the development of fetal vasculature in the labyrinthine layer of the placenta.

The “two-step” hypothesis of PE pathogenesis (35) suggested that high blood pressure and urine protein levels are just symptoms of “the second step,” while deficient trophoblast invasion and impaired SA remodeling in “the first step” is the fundamental cause of PE. In addition, good fetal vascular development and a normal fetal-maternal vascular network are the basis of placenta perfusion adequacy (36). In this study, we detected aberrant development of the fetal-maternal vascular network, characterized by a decreased number of vascular branches and narrowed vessel lumen, and deficient trophoblast invasion and impaired SA remodeling in MT after LPS injection. Based on our data, we thought that the established PE-like rat model could simulate the pathogenesis of clinical PE to a large extent and meet the needs of our follow-up experiments.

Abnormal macrophage polarization is related to many pregnancy complications, such as PE, premature birth and RPL (8, 12, 15). Consistent with previous results (12, 14, 15), we found the aberrant polarization of macrophages (increased M1 and decreased M2) in the MT of PE-like rats, which corresponded with the fact that LPS can induce macrophage polarization to M1 subsets (34). We also detected reduced Tim-3 expression in DMs, increased pro-inflammatory and decreased anti-inflammatory cytokines in MT of PE-like rats. Zhang et al. (37) also found Tim-3 expression on human CD14^+^ monocytes/macrophage was declined upon LPS stimulation. However, the reason why the expression of Tim-3 in DMs decreased after the LPS administration is not clear.

Previous findings simply indicated that, in certain diseases, such as inflammatory bowel disease (20), sepsis (38), and cancers (21), an imbalance of M1/M2 was present and related to the altered Tim-3 expression on macrophages. However, the mechanisms for this process have not been elucidated clearly. Yang et al. (38) thought that Tim-3 inhibited LPS/TLR4-mediated NF-κB activation by increasing PI3K/AKT phosphorylation and A20 activity, which promoted M1 subtype polarization. Cross-talk between the Tim-3 and TLR4 pathways makes TLR4 an important contributor to Tim-3-mediated negative regulation of macrophage polarization. Sun et al. (39) reported that miR-330-5p could directly regulate Tim-3 expression. IL-27 and the tumor microenvironment were also involved in modulating Tim-3 expression (21, 40). However, unlike other negative immunoregulatory molecules, such as PD-1 (41), Tim-3 has no inhibitory motif because its intracellular domain is relatively shorter. Therefore, studies about the upstream and/or downstream molecules (signalings) were very rare. Herein, the underlying mechanism how LPS administration affects Tim-3 expression in DMs still needs further investigation.

Scientific evidence has suggested that Tim-3 binding with its ligand Gal-9 could induce immune tolerance by inhibiting the function of T cells and then maintain immune tolerance (42). It was thought that the Tim-3/Gal-9 signaling pathway plays critical roles in the field of transplantation, autoimmune disease and maternal-fetal immune tolerance (43, 44). Gal-9 administration improves immune complex induced arthritis by regulating functions of T cells and macrophages (45, 46). Gal-9 has been shown to diminish the clinical severity of lupus in lupus-prone mice model (47). However, whether administration of Gal-9 could alleviate PE-like manifestations by regulating DM polarization has yet to be addressed. To verify whether this pathway could switch macrophage polarization to M2 subsets in the PE-like rat model, we treated the rats which received LPS with Gal-9.

Basing on the development of the placenta in rats (48, 49), trophoblast invasion accompanied by choriovitelline placenta formation at E7 gives rise to the definitive placenta. Around E12, trophoblast cells invade the endovasculature to replace vascular endothelial cells, also known as SA remodeling, and the choriovitelline placenta develops into the chorioallantoic placenta at the same time. Based on the characteristics of placental development and our previous study (50), we chose E16 as the last time to inject Gal-9. Finally, we conducted the experiments with Gal-9 administration at E7, E12, and E16, respectively. For the intervention dosage, there has been no similar study. The Gal-9 dosage we used was referred to previous researches about blocking several negative co-stimulatory molecules, such as PD-1 and Tim-3 (23, 51). However, in some studies, the PD-1 and Tim-3 antibodies were even used at high doses of 500 mg, 250 mg and 250 mg at different time points (44, 52, 53). Taking into account the whole project and the effect of drug absorption, we finally chose the dosages of Gal-9 administration (500 μg at E7, 250 μg at E12 and 250 μg at E16, respectively) via intraperitoneal injection in this study. Our results showed that Gal-9 alleviated the PE-like manifestations, mainly characterized by decreased blood pressure and urine protein, less liver and kidney damage, and favorable trophoblast invasion and SA remodeling. These results suggested that Gal-9 had protective effects in PE-like rats.

In addition to having effects on the mother, PE can threaten the fetus, leading to some disorders, such as FGR (54). In our study, FGR was also observed in rats treated with LPS, even accompanied with hind limb deformation, which was consistent with published results (55). Comparing the development of the placenta in all three groups, we found an irregular shape and a bleak color signifying placental ischemia and the fetal absorption in PE-like models, similar to a previous study (31). When fetal development and growth potential initiated by genes are inconsistent, FGR will occur (56). Also, previous evidence showed that abnormal maternal inflammatory response was related to FGR (57). Significantly increased pro-inflammatory cytokines and chemokines were detected systemically in the placenta of the pregnant women suffering from FGR (57, 58). It was generally thought that FGR resulted from uteri-placental insufficiency, which would lead to placental ischemia and poor placentation (59). Our data showed that reduced branches of the placental chorionic plate, less number of fetal vessels and the narrow lumen were observed in PE-like rats, which indicated impaired fetal-maternal vascular network development. Poor placentation can release a complex mix of factors, such as pro-inflammatory cytokines and soluble fms-like tyrosine kinase 1 (sFlt-1), into the maternal circulation, inducing systemic endothelial dysfunction, hypertension, proteinuria and glomerular endotheliosis (60–62). Insufficient blood supplement and systemic maternal inflammatory response may contribute to FGR, together with local pro-inflammatory microenvironment. However, these changes in the placenta and fetus were not observed in the rats receiving Gal-9, and mushroomed and fresh-colored placentas developed normally like the control group, suggesting that Gal-9 also had protective effects on the fetus.

Interestingly, the Gal-9 expression at the maternal-fetal interface was decreased in the LPS-treated group and was rescued after exogenous administration of Gal-9 arriving at the similar level as observed in the control group. Moreover, Gal-9 protein was mainly localized at one pole of embryo implantation at E9 and in the spongiotrophoblast layer and decidua at E20, which was consistent with other study (63). It is known that the trophoblast cells in the spongiotrophoblast layer have the invasive function. Some literatures have reported that Gal-9 had a relationship with the invasion of trophoblast cells (64). We speculated that the exogenous Gal-9 first regulated DM polarization shift to M2, then a cross-talk between DMs and the trophoblast cells promoted Gal-9 expression and thus improved trophoblast invasion. However, the exact mechanism remains unclear and needs further investigations.

Previous studies reported that the Tim-3/Gal-9 signaling pathway could maintain normal pregnancy via regulating decidual NK cells (27, 65), but the effects in DMs were not well-demonstrated. Our data showed that administration of Gal-9 shifted macrophages to M2 polarization and increased Tim-3 expression in DMs, accompanying with reduced pro-inflammatory and increased anti-inflammatory cytokines. A recent study suggested that Gal-9, as novel pathogen recognition molecular, can recognize LPS on Gram-negative bacteria and then induce neutrophil Tim-3-mediated bacterial killing (66). In sepsis, Tim-3 signaling in macrophages also inhibited LPS/TLR4-mediated pro-inflammatory cytokine production (38). It was also demonstrated that Gal-9 inhibits TLR7/TLR9-mediated lupus in mouse models by modulating plasmacytoid DCs and B cells, and protects kidney from immune complex-induced damage (67). These data suggest that a cross-interaction between LPS and Tim-3/Gal-9 signaling might be present in this study, which needs further validation.

It should be noted that Gal-9 might also regulate the function of other immune cells except for macrophages. Early studies showed that Gal-9 caused Th1 cell death and tolerance by interacting with Tim-3 (68). The Tim-3/Gal-9 pathway was involved in modulation of neutrophil degranulation and NADPH oxidative activity, promoting neutrophil-mediated Gran-negative bacterial killing (66). Zhao et al. (22, 38) demonstrated that blocking Tim-3 could exacerbate sepsis, while over-expressing Tim-3 or administering Gal-9 attenuated sepsis and significantly improved survival. Besides, several researches indicated that decidual NK cells, CD4^+^T cells and CD8^+^T cells also expressed Tim-3 and subsequently participated in the immune tolerance during pregnancy (69). Tim-3^+^decidual NK cells displayed features of immune tolerance, characterized by higher level of Th2 cytokine, lower level of Th1 cytokine and lower cytotoxicity. Blocking Tim-3/Gal-9 pathway by administration of anti-Tim-3 mAb could inhibit the transformation of periphery NK cells to decidual NK cells (27, 51). At the same time, Tim-3 signaling could also regulate the function of CD4^+^T cells and CD8^+^T cells at maternal-fetal interface, switching it to a pregnancy maintenance beneficial for immune microenvironment (44, 52). The above-mentioned studies indicate that the regulatory effects of Tim-3 on various decidual immune cells, including DMs, may collectively contribute to the maintenance of a normal pregnancy. Whether abnormal Tim-3 expression on NK cells or T cells is present in our rat model needs further investigations.

In addition, there are still some limitations and shortcomings in this study. First, the specific intracellular signaling pathway regulated macrophage polarization by Tim-3/Gal-9 and the upstream signalings (molecules) affected Tim-3 expression in DMs still require further investigations. Second, in this study, we solely focused on the effects of macrophages but ignored other immune cells. Numerous immune cells, cytokines, and signal molecules from fetus, mother and placenta work in concert to protect the fetus from immunological recognition and rejection. Finally, the findings from this study came from the rat models, which need further verification on PE patients.

**Figure 10:**
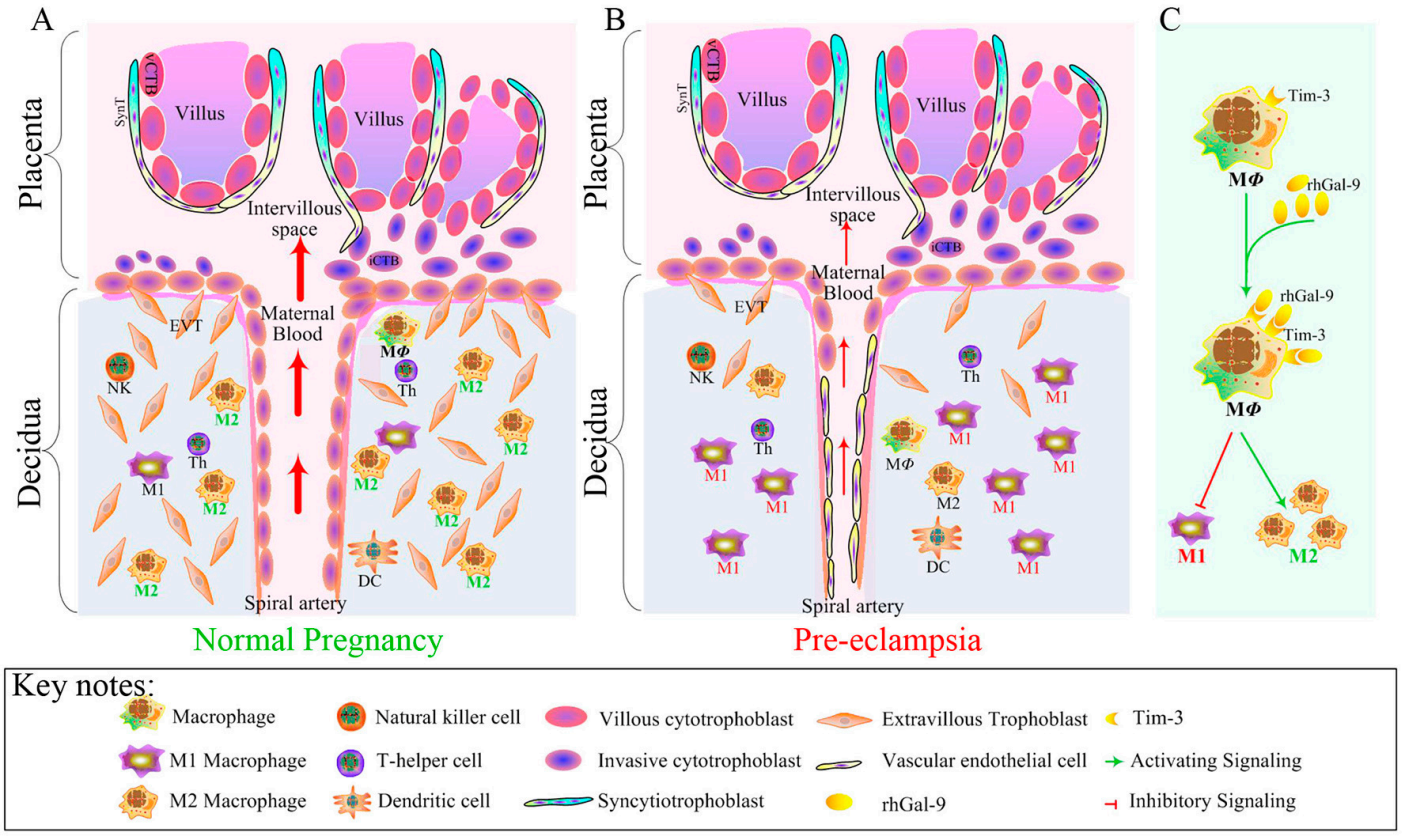
Tim-3-mediated harmonious dialogue at the maternal-fetal interface guarantees a smooth gestation by inducing M2 DM polarization. During normal pregnancy, the polarization of DMs is biased to M2 subsets to induce immune tolerance and promote trophoblast cell invasion and SA remodeling **(A)**, which are essential for the establishment and maintenance of a successful pregnancy. Aberrant expression of Tim-3 in DMs lead to Tim-3/Gal-9 signal pathway dysfunction, mediating the polarization of macrophage-biased to M1 subsets. The immune tolerance at the maternal-fetal interface was broken. Trophoblast cell invasion insufficiency and SA remodeling impairment ultimately lead to PE **(B)**. After exogenous administration of Gal-9, the expression of Tim-3 in DMs was reversed, and the polarization of DMs biased to M1 was suppressed but a shift to M2 was promoted **(C)**, which contributed to inducing immune tolerance.

According to the present data, we concluded that the decreased Tim-3 expression in DMs led to Tim-3/Gal-9 signaling pathway dysfunction, mediating the polarization of macrophages biased to M1 subsets. Then, the immune tolerance at the maternal-fetal interface was broken. Subsequently, the trophoblast cell invasion was insufficient and SA remodeling was impaired, which was the first step of the ‘two-stage theory’ of the pathogenesis of PE. However, after exogenous administration of Gal-9, altered Tim-3/Gal-9 pathway was reversed. Meanwhile, Tim-3 expression in DMs and Gal-9 at the maternal-fetal interface were increased. The polarization of macrophages was dominated by M2 subsets, which induced immune tolerance essential for a successful pregnancy (Figure 10). Thus, altered function of the Tim-3/Gal-9 axis is one primary cause of macrophage polarization imbalance that leads to PE. The *in vivo* study showed that Gal-9 had beneficial effects on the maternal and fetal development in the LPS-induced PE-like rat model. What is interesting is that the changes in our study are significant at E9, in the early stage of pregnancy, which may suggest that the earlier the Tim-3/Gal-9 pathway is treated, the better the pregnancy outcome will be. Therefore, our study provides not only ideas to elucidate the underlying pathogenesis of PE but also a new target for therapeutic strategies.

## Author Contributions

A-HL conceived and designed experiments. Z-HL and L-LW conducted all of the experiments and analyzed data. Z-HL prepared the manuscript draft. L-LW, HL, KM, X-BH, GM, and A-HL critically reviewed and edited the manuscript. A-HL supervised the project and, together with Z-HL and L-LW edited this final version of the manuscript.

### Conflict of Interest Statement

The authors declare that the research was conducted in the absence of any commercial or financial relationships that could be construed as a potential conflict of interest.
