# Supplementary material for: Galectin-9 Alleviates LPS-Induced Preeclampsia-Like Impairment in Rats via Switching Decidual Macrophage Polarization to M2 Subtype
Source: Front Immunol. 2019 Jan 10;9:3142. doi: 10.3389/fimmu.2018.03142 (PMC6335255; doi:10.3389/fimmu.2018.03142)
Supplement: Supplementary file 1 [file Data_Sheet_1.pdf]

Supplementary Material

Galectin-9 alleviates LPS-induced preeclampsia-like impairment in rats via switching decidual macrophage polarization to M2 subtype

Zhi-Hui Li[1†], Li-Ling Wang[1†], Hong Liu[1], Kahinho P. Muyayalo[1], Xiao-Bo Huang[1], Gil Mor[1,2], Ai-Hua Liao[1]*

[1] Family Planning Research Institute, Center for Reproductive Medicine, Tongji Medical College, Huazhong University of Science and Technology, Wuhan, China, [2] Reproductive Immunology Unit, Department of Obstetrics Gynecology and Reproductive Science, Yale University School of Medicine, New Haven, CT, United States

*** Correspondence:** Ai-Hua Liao, MD, PhD: E-mail:aihua_liao@sina.com

[†]These authors contributed equally to this work.

1 Supplementary Tables

Table S1

Antibodies used for immunohistochemistry (IHC), immunofluorescence (IF) and western blot (WB)

Antibody	Host	Dilution	Manufacturer	Cat#
anti-Cytokeratin 7 [EPR17078] (CK7)	Rabbit mAb	1:200	abcam, UK	ab181598
anti-alpha smooth muscle actin (α-SMA)	Goat pAb	1:200	abcam, UK	ab21027
anti-Laminin antibody	Rabbit pAb	1:200	abcam, UK	ab11575
anti-CD68 [ED1]	Mouse mAb	1:200	abcam, UK	ab31630
CCR7 antibody	Goat pAb	1:50	Novus, USA	NB 100-712
anti-liver Arginase antibody(Arg1)	Goat pAb	1:50	abcam, UK	ab60176
anti-Tim 3 antibody	Rabbit pAb	1:100	abcam, UK	ab185703
anti-Gal-9 antibody	Rabbit pAb	1:100	abcam, UK	ab69630
DyLight 488 AffiniPure Goat anti-Mouse IgG(H+L)	Goat pAb	1:200	abcam, UK	A23210
DyLight 594 AffiniPure Goat anti-Rabbit IgG(H+L)	Goat pAb	1:200	Abbkine, China	A23420
Donkey Anti-Rabbit IgG H&L (Alexa Fluor® 594)	Donkey pAb	1:500	abcam, UK	ab150076
Donkey Anti-Goat IgG H&L (Alexa Fluor® 488)	Donkey pAb	1:500	abcam, UK	ab150129
β-actin(C4)	Mouse mAb	1:2000	Santa Cruz, USA	sc-4778
goat anti-rabbit IgG-HRP	Goat pAb	1:1000	Santa Cruz, USA	sc-2004
donkey anti-goat IgG-HRP	Donkey pAb	1:1000	Santa Cruz, USA	sc-2020
anti-mouse IgG-HRP	Goat	1:1000	PTG, USA	SA00001-1

Table S2

Primer sequences for quantitative real-time PCR

Target	Forward primer	Reverse primer
β-actin	CTGAACCCTAAGGCCAACCG	GACCAGAGGCATACAGGGACAA
Arg1	TATCGGAGCGCCTTTCTCTA	ACAGACCGTGGGTTCTTCAC
iNOS	TCCTCAGGCTTGGGTCTTGTTAG	TTCAGGTCACCTTGGTAGGATTTG
IL-10	GAGAGAAGCTGAAGACCCTCTG	TCATTCATGGCCTTGTAGACAC
TGF-β	GGCACCATCCATGACATGAACCG	GCCGTACACAGCAGTTCTTCTCTG
TNF-α	GCCTCTTCTCATTCCTGCTC	CCCATTTGGGAACTTCTCCT
IL-1β	ATCTCACAGCAGCATCTCGACAAG	CACACTAGCAGGTCGTCATCATCC

Table S3

SBP profiles (mmHg) in different groups

Embryonic Day (E)	saline group (n=7)	LPS group (n=7)	LPS + Gal-9 group (n=5)
0	99.11±0.38	96.83±0.35	97.93±0.62
3	100.42±0.32	98.53±0.33	101.81±0.93
6	101.56±0.37	119.95±1.38 **	126.19±1.05$^{\triangle\triangle}$
8	102.26±0.91	120.57±1.17 $^{**}_{\#\#}$	109.02±0.87
10	101.56±0.73	123.71±0.78 $^{**}_{\#\#}$	104.99±1.79
12	101.99±0.69	123.44±0.38 $^{**}_{\#\#}$	102.90±0.44
14	102.82±0.69	126.00±0.68 $^{**}_{\#\#}$	103.92±0.80
16	101.28±0.49	121.61±0.56 $^{**}_{\#\#}$	101.51±0.48
18	99.98±0.43	125.73±0.61 $^{**}_{\#\#}$	102.06±0.64

$^{\triangle\triangle}p < 0.01$ LPS+Gal-9 vs. saline group at E6;
$^{**}p < 0.01$ LPS vs. saline pregnancy group at the corresponding embryonic day;
$^{\#\#}p < 0.01$ LPS vs. LPS+Gal9 group at the corresponding embryonic day.

Table S4

24h urinary protein level (mg/24h) in different groups

Embryonic Day (E)	saline group (n=7)	LPS group (n=7)	LPS + Gal-9 group (n=5)
0	2.23 ±0.08	2.25±0.05	2.11±0.04
3	2.16±0.03	2.30±0.05	2.39±0.05
6	2.72±0.62	4.13±0.04 **	4.29±0.07▲▲
9	2.37±0.06	4.57±0.06 **##	3.02±0.09
12	2.41±0.04	4.55±0.06 **##	3.19±0.08
15	2.49±0.08	5.49±0.05 **##	2.64±0.04
18	2.76±0.06	5.78±0.03 **##	2.85±0.04

△△$p < 0.01$ LPS+Gal9 vs. saline pregnancy group at E6;
**$p < 0.01$ LPS vs. saline pregnancy group at the corresponding embryonic day;
##$p < 0.01$ LPS vs. LPS+Gal9 group at the corresponding embryonic day.

Table S5

Fetal length (cm) and wet fetal wet weights (g) in different groups

Embryonic Day (E)	saline group (n=7)	LPS group (n=7)	LPS + Gal-9 group (n=5)
Fetal length (cm)	4.31±0.05	3.20±0.03 ** ##	4.05±0.03
Fetal Weight (g)	4.61±0.06	3.07±0.04 ** ##	4.20±0.06

** $p < 0.01$ LPS vs. saline pregnancy group at E20;

$p < 0.01$ LPS vs. LPS+Gal9 group at E20.

2 Supplementary Figures

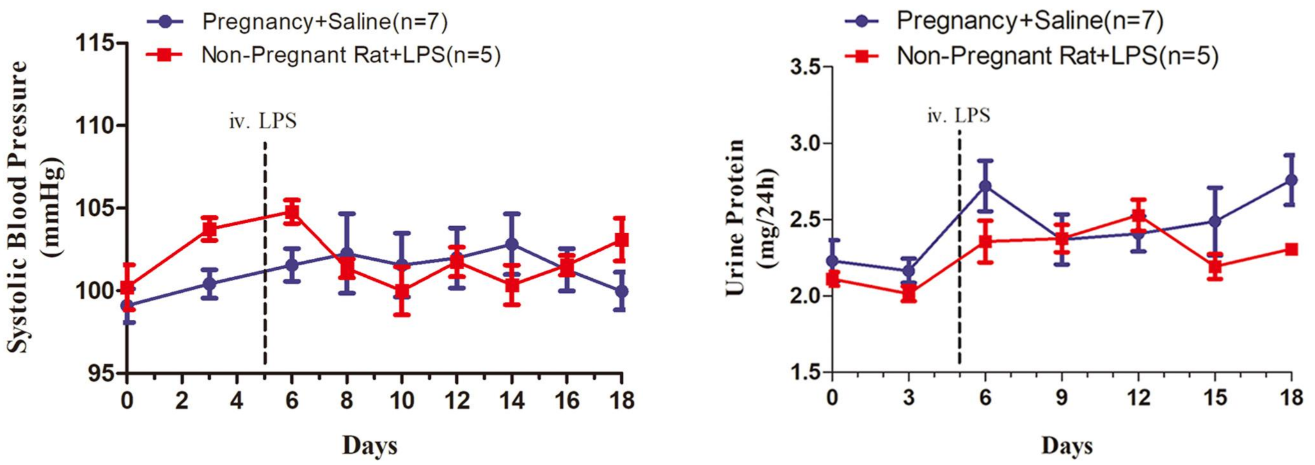

Supplementary Figure 1. Mean SBP and 24 h urinary protein in non-pregnancy group. Noted that significant increase of SBP and albuminuria was not observed in non-pregnant rats.

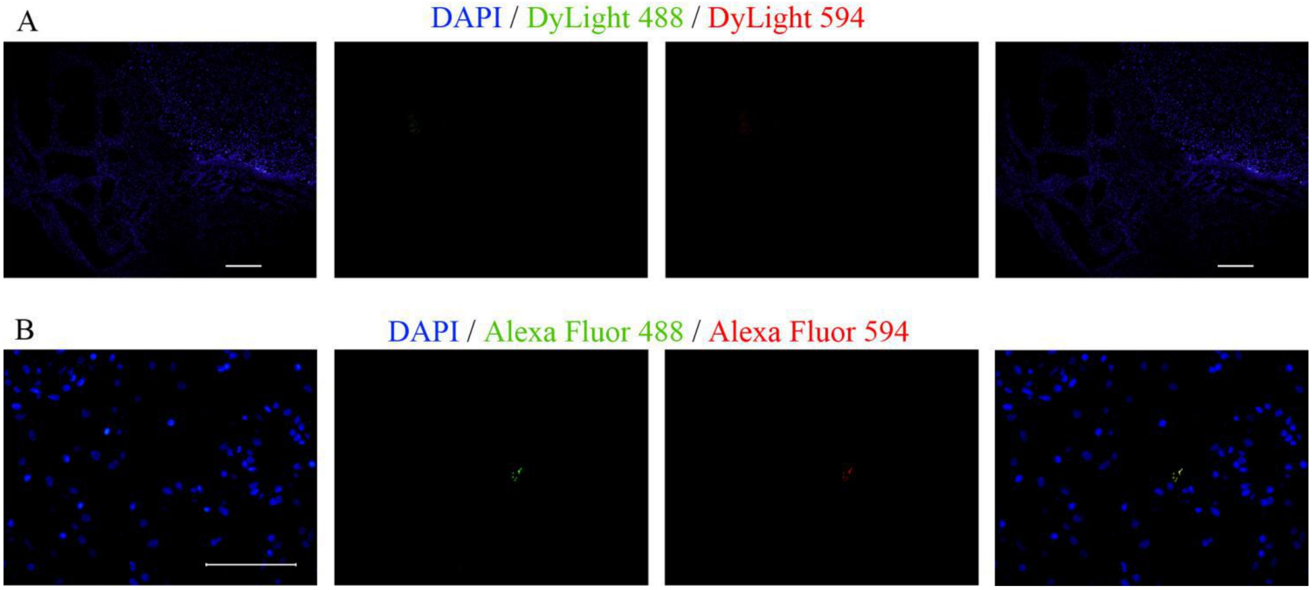

Supplementary Figure 2. Negative control images for the primary antibodies used in this study. (A) Negative control images for CK7 and α-SMA defaulted, only secondary antibodies of DyLight 488/594 were added. Bar = 500μm. (B) Negative control images for CD68, CCR7, Arg1 and Tim-3 defaulted, only secondary antibodies of Alexa Fluor 488/594 were added. Bar = 100μm.

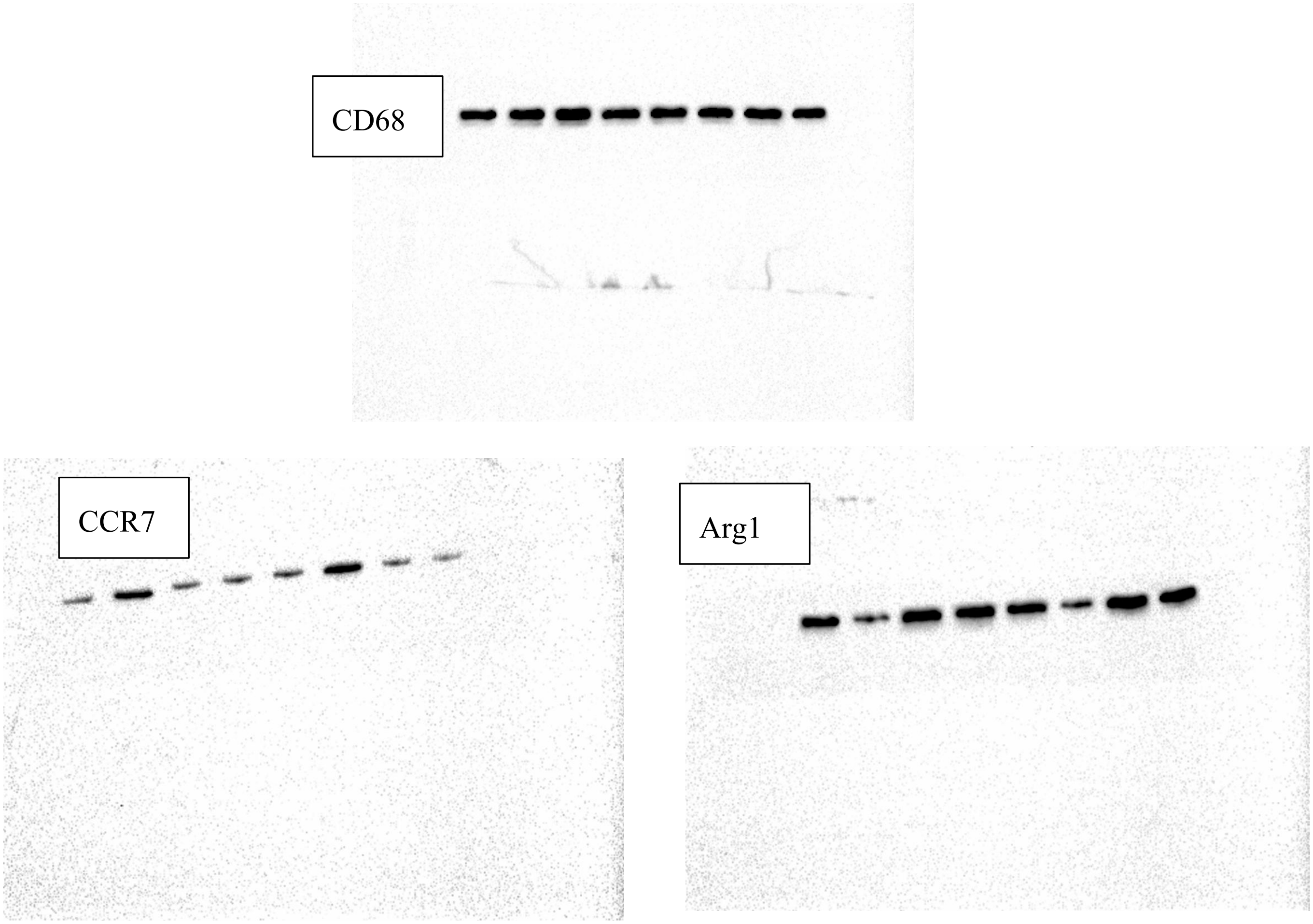

Supplementary Figure 3. Western blots of the entire original PVDF for CD68, CCR7 and Arg1 in the decidua in different groups. Detected the molecule repeatedly in one experiment and three independent experiments totally.

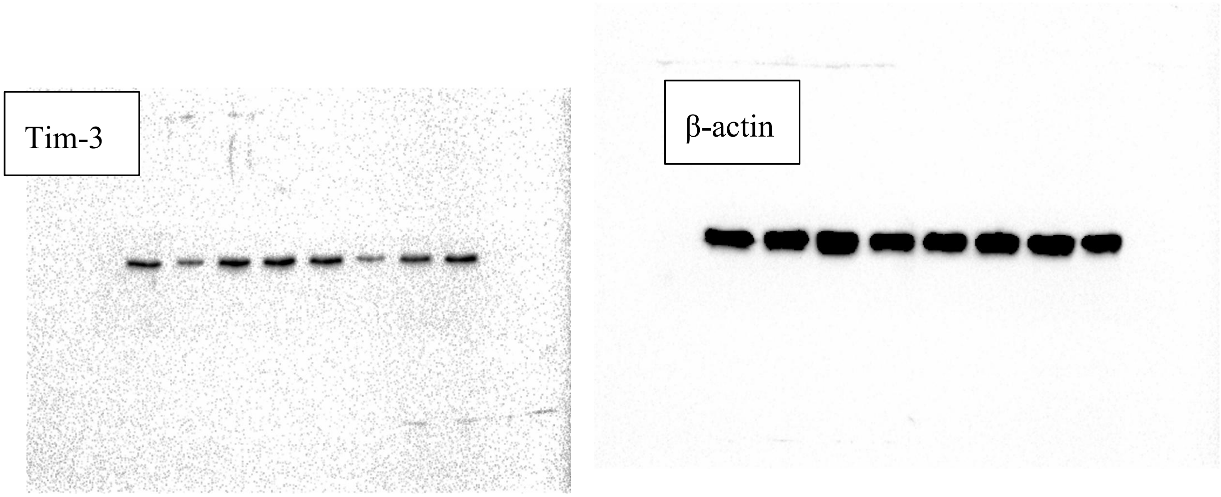

Supplementary Figure 4. Western blots of the entire original PVDF for Tim-3 and β-actin in the decidua in different groups. Detected the molecule repeatedly in one experiment and three independent experiments totally.

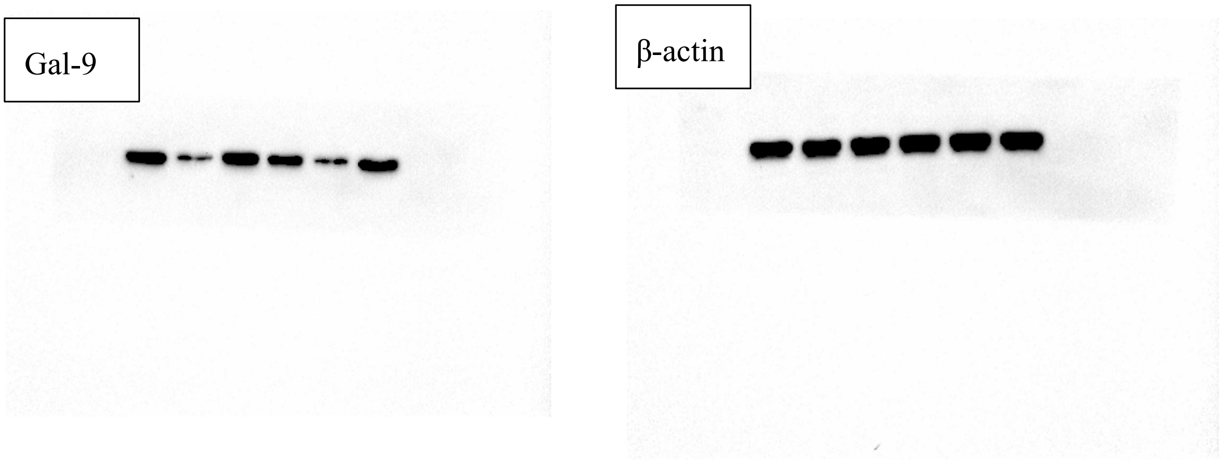

Supplementary Figure 5. Western blots of the entire original PVDF for Gal-9 and β-actin in the decidua in different groups.

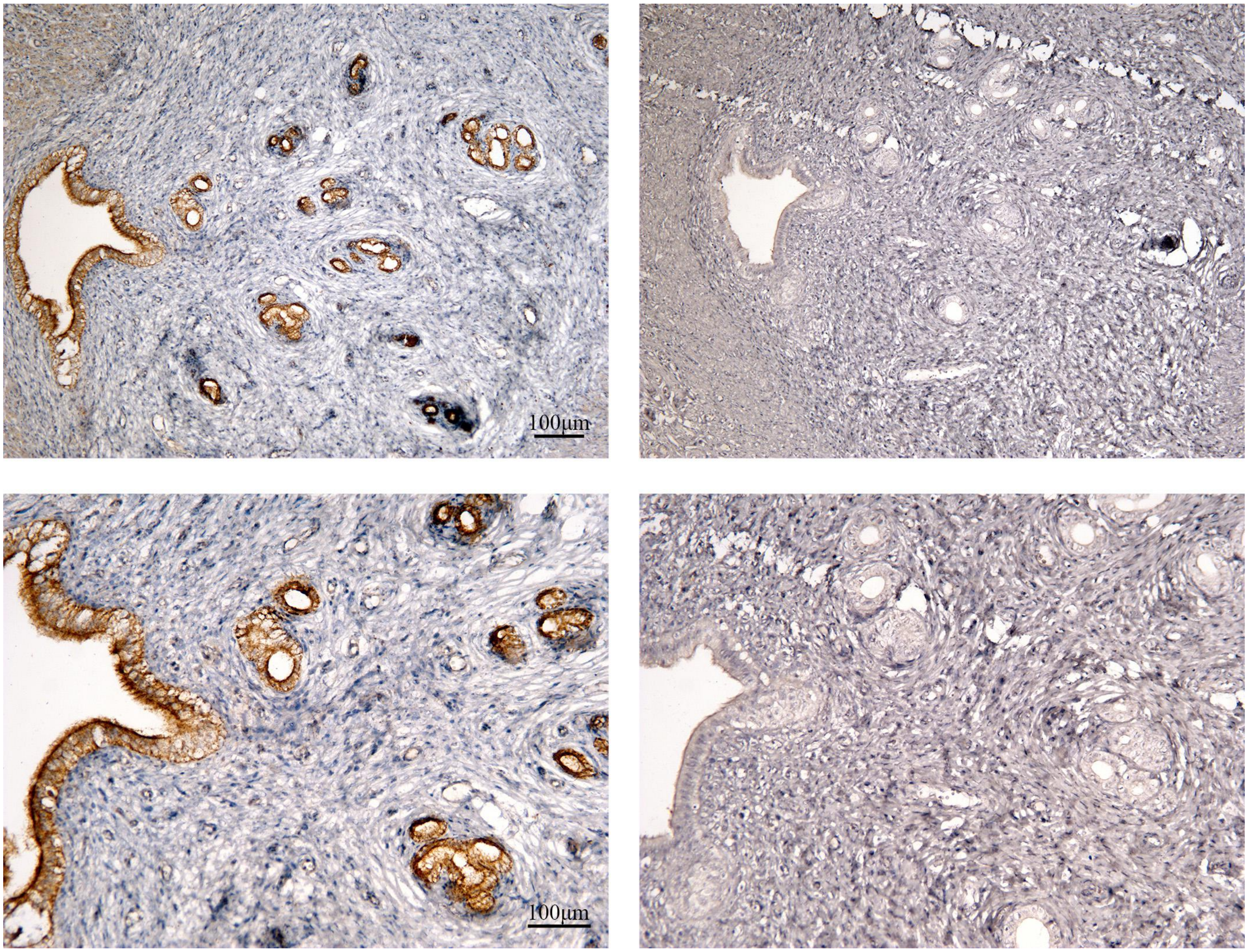

Supplementary Figure 6. Immunohistochemistry for Gal-9 in non-pregnancy groups. Noted that the expression of Gal-9 in non-pregnancy rat uteri was mainly localized on endometrial and glandular epithelium. The right panels are the negative controls. Bar = 100μm.
